# Global, regional, and national burden of laryngeal cancer in middle-aged and older adults from 1990 to 2021: an analysis of age and sex differences and attributable risk factors

**DOI:** 10.3389/fpubh.2025.1601029

**Published:** 2025-05-30

**Authors:** Hongxia Xiao, Hui Wang, Hui Zhang, Shanying Wu, Bo Yu

**Affiliations:** ^1^Department of Otolaryngology, Linyi Central Hospital, Linyi, China; ^2^Department of Otolaryngology, Head and Neck Surgery, The Second Hospital of Weihai City, Weihai, China; ^3^Department of Otolaryngology, Head and Neck Surgery, The Second Hospital of Dalian Medical University, Dalian, China

**Keywords:** laryngeal cancer, global burden of disease, middle-aged, older adult, risk factors

## Abstract

**Background:**

Laryngeal cancer (LC) is a prevalent head and neck tumor, yet few studies quantify its disease burden and trends in risk factors of the age subgroup of middle-aged and older adult people (MAOP).

**Methods:**

Based on Global Burden of Disease 2021 (GBD 2021) data, this study comprehensively assessed trends of disease burden in the age subgroup of LC in MAOP across 204 countries and regions from 1990 to 2021, covering incidence, prevalence, mortality, and disability-adjusted life years (DALYs). The estimated annual percentage change (EAPC) and joinpoint regression model were used to analyze the trends of disease burden and the changes in disease burden attributed to tobacco, alcohol, and occupational risk factors.

**Results:**

Worldwide, the number of LC in MAOP prevalence, incidence, mortality, and DALYs totaled 887,500, 163,300, 97,500, and 2,284,800 in 2021. Over the previous 32 years, the rates of these indicators declined, with EAPC of −0.62 (−0.69 to −0.55), −0.95 (−1.04 to −1.87), −1.52 (−1.6 to −1.43), and −1.69 (−1.79 to −1.59), respectively. Regions with high Sociodemographic Index (SDI) showed high prevalence and morbidity. Still, they had low mortality and DALY rates, whereas areas with low SDI, such as Sub-Saharan Africa, experienced high mortality and DALY rates. Central Europe, a high-middle SDI region, was a “hotspot” for laryngeal cancer. Before the age of 75, males showed a more significant burden of disease indicators, with a peak incidence rate of 28.05 (23.93, 30.47) per 100,000 individuals between the ages of 85 and 90. However, the 85–89 and 90–94 age groups saw rising prevalence rates. The main risk factor for LC-related deaths and DALY in MAOP was tobacco exposure, particularly in low-middle SDI regions.

**Conclusion:**

In the last 32 years, the burden of LC in MAOP has declined globally, yet age-related disparities and SDI stratification persist. Men aged before 75 and after 85 need to be targeted as key groups for prevention and control. Tobacco exposure was the leading risk factor, especially in low-middle SDI regions. These findings highlight the critical necessity for tailored public health strategies and policies.

## 1 Introduction

Due to its unique anatomical features, laryngeal cancer, a cancer affecting the head and neck region, represents a significant global health challenge by threatening patients’ respiratory, swallowing, and speaking abilities (1). Epidemiological statistics from the GBD 2019 study revealed that the total number of new cases of LC worldwide was 209,000, with males comprising 181,000 cases and females accounting for 28,500 instances (2). The epidemiologic profile of laryngeal cancer demonstrates significant demographic and geographic heterogeneity, with countries exhibiting relatively low SDI and MAOP male demographics experiencing the highest incidence of laryngeal cancer. Age-standardized mortality rates in developing countries (such as Pakistan, Seychelles, and Cuba) exceed 5 per 100,000, while those in developed countries (such as Japan, Singapore, and New Zealand) are below 0.5 per 100,000 (3). The disease burden is exceptionally high in MAOP. At present, the aging process is accelerating significantly around the world. According to the report by the World Health Organization, the population over 60 will grow from 1 billion to 1.4 billion from 2020 to 2030 (4).

However, regional differences still exist in the current prevention and control of LC. Statistically, one-third of the world’s poorest people have limited surgical coverage (3.5%), and there is a lack of consensus on the definition of surgery and surgical care (5). Numerous LMICs struggle with inadequate infrastructure, physical resources, and workforce necessary for delivering essential surgical care, challenging the idea that surgical issues are swiftly resolved. Furthermore, LMICs encounter substantial disparities in access to cancer treatment drugs. In many wealthy countries, patients can access cancer medications, including new treatments, without paying a substantial amount out of pocket. On the other hand, 40% of conventional chemotherapy medicines are only accessible to patients in LMICs (6).

As the immune system undergoes a gradual decline, compounded by long-term risk factors like tobacco use and alcohol intake (7), along with the significant impact of human papillomavirus (HPV) infection (8), the MAOP has become a high-risk group for LC. Despite noteworthy advancements in laryngeal cancer research within the biomedical field and the emergence of sophisticated analytical techniques, this malignancy continues to impose a substantial burden on the health of humans, particularly the older adult population. Middle-aged and older adult patients, often afflicted with underlying chronic diseases, frequently exhibit unsatisfactory prognoses, with a 5-year relative survival rate of approximately 61% (9). However, the burden of LC and its trends in MAOP, especially across distinct age subgroups, remain subjects of current understudies.

The GBD 2021 database is a global public health research project resource with a wide range of impact and scale. It systematically integrates data on 371 diseases, injuries, and risk factors from more than 200 countries and territories (10). The GBD database covers incidence, prevalence, mortality, DALYs for diseases and injuries, and attribution analysis of associated risk factors. The project provides a critical scientific basis for global public health policy, disease prevention and control development, and healthcare resource distribution, which helps to promote global health equity.

Therefore, based on GBD 2021 data, this study provides an in-depth analysis of epidemiologic characteristics, risk factors, projections, and trends in the burden of disease of LC in MAOP, especially in the age subgroups. The study is essential for formulating strategies for preventing and controlling laryngeal cancer and promoting the equitable distribution of medical resources. The study aims to achieve the following primary objectives: (1) to quantitatively analyze the spatial and temporal distributions and age subgroup distribution characteristics of the prevalence, incidence, mortality, and DALY rates of LC in MAOP from 1990 to 2021; (2) to evaluate the impact of significant risk factors across various age groups, genders, and regions; and (3) to propose stratified prevention and control strategies for LC in MAOP.

## 2 Materials and methods

### 2.1 Data sources

This research utilized the GBD 2021 database, which covers the epidemiological information of 371 diseases and injuries and 88 risk factors in 204 countries and regions worldwide between 1990 and 2021 (10). The database details, such as the construction method, index definition, and data collection channels, have been systematically discussed and explained in relevant academic literature. This study extracted epidemiologic data on LC in MAOP from this database for the world, five SDI regions, 21 GBD-defined regions, and 204 countries for people aged 55 years and older. The study covered four leading disease indicators: prevalence, morbidity, mortality, and DALY values and rate values, and included secondary risk factors: alcohol use, occupational risks, and tobacco use. The study’s dataset was sourced from the Global Health Data Exchange query tool.^1^

### 2.2 Research type and design

Based on the type of secondary data analysis, this study was divided into subgroups according to age. The disease burden indicators and attribution risk factors of LC in MAOP at the global, regional, and national levels in the GBD 2021 database were intensely discussed. The research subjects are individuals aged 55 and older, further divided into nine age subgroups (55–59, 60–64, 65–69, 70–74, 75–79, 80–84, 85–89, 90–94, 95+). Additionally, this study analyzes the association between SDI and disease burden.

### 2.3 Statistical analysis

#### 2.3.1 Descriptive analysis

This study used descriptive analytic metrics covering numbers and rates of incidence, prevalence, mortality, and DALY for LC in MAOP. Mortality and DALY rates due to secondary attributable risk factors for LC in MAOP were also explored. The trends of disease burden indicators, including incidence, mortality, and DALY, were evaluated by estimating the EAPC, 95% confidence intervals (CIs), and their corresponding *p*-values. The formula for calculating EAPC is: EAPC = 100 × [exp(*β*) − 1] (11). Statistically significant increasing burden levels were identified when the EAPC point estimate and the lower confidence boundary exceeded zero. Conversely, an EAPC value with its upper CI limit below zero demonstrated a statistically significant decreasing pattern. A 95% CI range of 0 is considered to be relatively stable.

#### 2.3.2 Correlation analysis

The SDI is a comprehensive indicator for measuring a country or region’s social and economic development. Based on SDI values, the world is divided into five levels: low SDI, low-middle SDI, middle SDI, high-middle SDI, and high SDI (12). This study explored the correlation between the prevalence, incidence, mortality, DALY rate, and SDI of LC in MAOP. Correlation coefficient (*r*) values were used to quantify the degree of linear correlation between SDI and the burden indicators of LC in MAOP. A positive association is evidenced when the r value exceeds the zero threshold, meaning the disease burden increases with rising SDI. A negative correlation is evidenced when the r value is below zero, meaning the disease burden increases with rising SDI.

#### 2.3.3 Joinpoint regression analysis

The joinpoint regression model was used to analyze the temporal evolution trends of risk factors attributed to mortality and DALY rates of LC in MAOP. Differences among regions, genders, and age groups were analyzed. This study implemented significance testing for joinpoint configurations through the Monte Carlo permutation method. The evolutionary trend of the time series was analyzed using the average annual percentage change (AAPC) and annual percentage change (APC) indicators (13). APC metric quantifies temporal dynamics within a specific period, whereas the AAPC can evaluate the overall change trend during the entire observation period. When the values of APC and AAPC are above zero, they suggest a rising pattern; conversely, when these indicators are under zero, they denote a declining tendency.

When the *p*-value falls below 0.05, it implies that the difference has statistical significance. The analyses were executed through R (version 4.4.2), the ggplot2 package, and Joinpoint software.

## 3 Results

### 3.1 Global level

**Figure 1:**
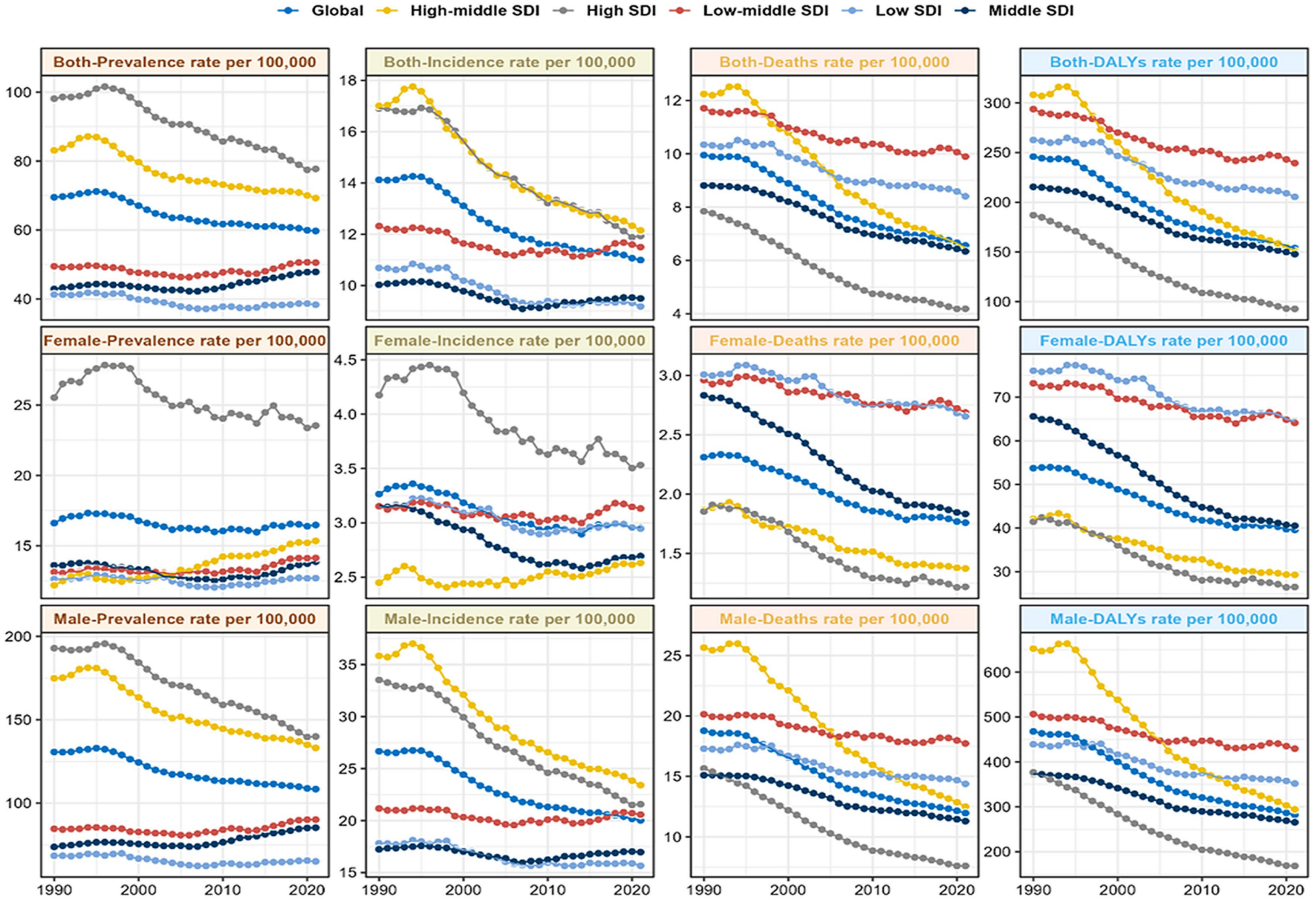
Disease trends by 5 SDI regions and gender for the prevalence rate, incidence rate, death rate, and DALYs rate from 1990 to 2021. DALYs, disability-adjusted life-year; SDI, sociodemographic index.

An overall decreasing trend in the rate of prevalence, incident, mortality, and DALYs of laryngeal cancer related to middle-aged and older adults was reported globally. Compared to the prevalence rate and incidence rate, the overall trend of mortality and DALYs rates was more positive (Figure 1). The prevalent cases increased from 466,713 cases (442,851 to 491,527) in 1990 to 887,580 cases (832,463 to 952,557) in 2021. The incident cases increased from 94,791 (90,077 to 99,538) in 1990 to 163,316 (151,965 to 175,890) in 2021. The rates of deaths and DALYs in 2021 were 6.56 (6.12, 7.7) per 100,000 population and 153.76 (143.31 to 165.63) per 100,000 population, with EAPC of −1.52(−1.6 to −1.43), and −1.69(−1.79 to −1.59), respectively (Figure 1 and Table 1).

**Table 1:** The Incidence and DALYs of LC in MAOP in 1990 and 2021, and the trends from 1990 through 2021.

Location	Incidence				1990–2021	DALYs				1990–2021
	1990		2021			1990		2021		
	Counts	Rate	Counts	Rate	EAPC	Counts	Rate	Counts	Rate	EAPC
	(95%UI)	pre 100,000 (95%UI)	(95%UI)	pre 100,000 (95%UI)	(95%CI)	(95%UI)	pre 100,000 (95%UI)	(95%UI)	pre 100,000 (95%UI)	(95%CI)
Global	94,791 (90,077, 99,538)	14.12 (13.42, 14.82)	163,316 (151,965, 175,890)	10.99 (10.23, 11.84)	−0.95***	1,650,951 (1,550,393, 1,753,792)	245.89 (230.91, 261.2)	2,284,843 (2,129,591, 2,461,289)	153.76 (143.31, 165.63)	−1.69***
					(−1.04 to −0.87)					(−1.79 to −1.59)
Male	83,040 (78,802, 87,797)	26.66 (25.3, 28.19)	140,040 (130,074, 151,216)	20.02 (18.59, 21.62)	−1.07***	1,457,568 (1,370,893, 1,555,963)	467.94 (440.12, 499.53)	1,973,496 (1,831,717, 2,140,379)	282.12 (261.85, 305.98)	−1.81***
					(−1.16 to −0.98)					(−1.92 to −1.71)
Female	11,751 (9,803, 12,760)	3.26 (2.72, 3.55)	23,276 (20,094, 26,886)	2.96 (2.55, 3.42)	−0.49***	193,384 (151,954, 215,932)	53.73 (42.22, 59.99)	311,347 (266,528, 369,129)	39.59 (33.89, 46.94)	−1.16***
					(−0.57 to −0.41)					(−1.25 to −1.08)
High SDI	31,517 (30,431, 32,518)	16.9 (16.32, 17.44)	41,153 (38,536, 43,117)	11.93 (11.17, 12.5)	−1.27***	348,603 (336,985, 360,624)	186.96 (180.72, 193.4)	319,644 (300,329, 335,581)	92.65 (87.05, 97.27)	−2.4***
					(−1.34 to −1.19)					(−2.51 to −2.29)
High-middle SDI	29,339 (27,985, 30,804)	17.01 (16.22, 17.85)	42,105 (37,986, 46,467)	12.15 (10.96, 13.4)	−1.3***	531,737 (504,934, 559,963)	308.21 (292.67, 324.57)	522,296 (473,196, 573,364)	150.66 (136.49, 165.39)	−2.61***
					(−1.4 to −1.2)					(−2.74 to −2.49)
Middle SDI	17,396 (15,933, 18,805)	10.02 (9.18, 10.83)	44,598 (39,812, 49,987)	9.49 (8.47, 10.64)	−0.29***	373,973 (341,951, 405,274)	215.47 (197.02, 233.51)	694,235 (623,988, 773,960)	147.75 (132.8, 164.72)	−1.36***
					(−0.38 to −0.19)					(−1.44 to −1.28)
Low-middle SDI	12,415 (10,670, 14,568)	12.32 (10.59, 14.45)	27,713 (24,981, 31,139)	11.5 (10.36, 12.92)	−0.26***	296,053 (253,853, 348,887)	293.7 (251.84, 346.12)	577,012 (519,043, 653,346)	239.34 (215.3, 271)	−0.69***
					(−0.35 to −0.18)					(−0.76 to −0.62)
Low SDI	3,986 (3,171, 4,892)	10.69 (8.5, 13.11)	7,532 (6,508, 8,645)	9.18 (7.93, 10.53)	−0.6***	97,982 (77,980, 120,328)	262.63 (209.02, 322.52)	168,518 (145,326, 195,214)	205.36 (177.1, 237.9)	−0.91***
					(−0.69 to −0.5)					(−1 to −0.83)
High-income Asia Pacific	3,879 (3,517, 4,223)	11.09 (10.06, 12.08)	5,570 (4,830, 6,209)	7.9 (6.85, 8.81)	−1.37***	34,009 (29,592, 37,825)	97.26 (84.63, 108.17)	29,225 (25,352, 32,612)	41.45 (35.96, 46.26)	−3.18***
					(−1.55 to −1.19)					(−3.35 to −3.02)
High-income North America	11,380 (10,971, 11,707)	19.64 (18.94, 20.21)	15,903 (15,005, 16,576)	14.13 (13.33, 14.73)	−1.43***	97,562 (93,994, 100,777)	168.42 (162.26, 173.97)	108,240 (102,557, 113,509)	96.18 (91.13, 100.87)	−2.13***
					(−1.54 to −1.32)					(−2.22 to −2.03)
Western Europe	21,332 (20,323, 22,143)	21.97 (20.93, 22.8)	21,710 (20,173, 23,124)	14.56 (13.53, 15.51)	−1.27***	260,229 (249,542, 270,502)	267.97 (256.96, 278.55)	178,824 (165,737, 190,279)	119.91 (111.13, 127.59)	−2.57***
					(−1.37 to −1.17)					(−2.72 to −2.43)
Australasia	408 (375, 445)	10.35 (9.53, 11.29)	500 (441, 560)	5.66 (4.99, 6.34)	−1.99***	5,860 (5,336, 6,461)	148.75 (135.44, 164)	5,017 (4,401, 5,613)	56.79 (49.82, 63.54)	−3.21***
					(−2.09 to −1.89)					(−3.33 to −3.09)
Andean Latin America	202 (175, 233)	6.03 (5.23, 6.95)	398 (310, 500)	4.01 (3.13, 5.04)	−1.5***	4,283 (3,721, 4,952)	127.61 (110.88, 147.55)	6,722 (5,220, 8,373)	67.86 (52.69, 84.52)	−2.26***
					(−1.77 to −1.23)					(−2.51 to −2)
Tropical Latin America	2,203 (2,113, 2,306)	14.55 (13.96, 15.23)	6,021 (5,619, 6,387)	13.59 (12.68, 14.42)	−0.21***	47,538 (45,606, 49,709)	313.96 (301.2, 328.3)	108,367 (101,244, 114,794)	244.63 (228.55, 259.14)	−0.77***
					(−0.29 to −0.12)					(−0.85 to −0.68)
Central Latin America	1,511 (1,452, 1,567)	11.14 (10.7, 11.55)	2,611 (2,293, 2,978)	6.1 (5.36, 6.96)	−2.43***	30,477 (29,286, 31,597)	224.59 (215.81, 232.84)	44,201 (38,828, 50,387)	103.35 (90.79, 117.82)	−2.98***
					(−2.58 to −2.27)					(−3.11 to −2.84)
Southern Latin America	1,491 (1,370, 1,623)	18.82 (17.3, 20.49)	1,595 (1,440, 1,751)	10.84 (9.78, 11.9)	−1.82***	28,594 (26,196, 31,186)	360.96 (330.7, 393.69)	23,701 (21,502, 25,923)	161.06 (146.11, 176.15)	−2.6***
					(−1.98 to −1.66)					(−2.74 to −2.45)
Caribbean	779 (712, 859)	18.08 (16.52, 19.93)	1857 (1,580, 2,197)	20.06 (17.07, 23.73)	0.43***	14,056 (12,849, 15,534)	326.14 (298.15, 360.44)	28,879 (24,606, 34,155)	311.92 (265.77, 368.91)	−0.03
					(0.32 to 0.53)					(−0.12 to 0.07)
Central Europe	5,324 (5,054, 5,635)	20.08 (19.06, 21.25)	7,506 (6,869, 8,178)	20.27 (18.55, 22.09)	−0.01	108,949 (103,485, 115,390)	410.81 (390.21, 435.1)	111,426 (102,019, 121,099)	300.92 (275.52, 327.05)	−1.1***
					(−0.09 to 0.07)					(−1.18 to −1.03)
Eastern Europe	8,924 (8,575, 9,282)	18.25 (17.54, 18.98)	8,230 (7,287, 9,210)	13.26 (11.74, 14.84)	−1.7***	182,860 (175,397, 189,861)	374 (358.74, 388.32)	129,518 (114,774, 145,363)	208.63 (184.88, 234.16)	−2.62***
					(−1.93 to −1.46)					(−2.87 to −2.37)
Central Asia	1,174 (1,119, 1,236)	14.67 (13.99, 15.46)	1,033 (922, 1,148)	7.1 (6.33, 7.89)	−2.46***	26,570 (25,321, 27,923)	332.2 (316.59, 349.13)	20,640 (18,340, 23,085)	141.86 (126.05, 158.66)	−2.92***
					(−2.68 to −2.24)					(−3.15 to −2.69)
North Africa and Middle East	3,630 (3,025, 4,247)	12.84 (10.7, 15.03)	9,253 (8,161, 10,535)	12.14 (10.71, 13.82)	−0.24***	75,018 (62,460, 87,921)	265.42 (220.99, 311.07)	131,355 (115,290, 149,765)	172.31 (151.23, 196.46)	−1.48***
					(−0.29 to −0.18)					(−1.53 to −1.43)
South Asia	14,696 (12,361, 17,356)	15.48 (13.02, 18.28)	34,234 (29,924, 39,190)	13.79 (12.05, 15.78)	−0.55***	355,251 (299,352, 419,721)	374.18 (315.3, 442.08)	709,622 (618,985, 816,799)	285.8 (249.29, 328.96)	−1.03***
					(−0.69 to −0.4)					(−1.15 to −0.9)
Southeast Asia	2,951 (2,543, 3,338)	6.97 (6.01, 7.88)	8,413 (7,255, 9,901)	7.34 (6.33, 8.64)	0.07**	61,962 (53,106, 70,400)	146.34 (125.42, 166.27)	138,645 (120,057, 162,545)	121.03 (104.8, 141.89)	−0.71***
					(0.03 to 0.11)					(−0.74 to −0.68)
East Asia	12,361 (10,203, 14,435)	8.3 (6.85, 9.69)	33,498 (26,538, 42,311)	8.54 (6.77, 10.79)	0.19*	256,101 (210,196, 301,356)	171.93 (141.12, 202.32)	396,915 (313,629, 500,534)	101.22 (79.98, 127.65)	−1.74***
					(0.04 to 0.34)					(−1.85 to −1.62)
Oceania	13 (10, 17)	2.71 (2.04, 3.49)	30 (23, 38)	2.39 (1.84, 3.12)	−0.53***	281 (208, 368)	58.4 (43.31, 76.48)	609 (463, 808)	49.35 (37.48, 65.49)	−0.62***
					(−0.61 to −0.46)					(−0.68 to −0.57)
Western Sub-Saharan Africa	815 (648, 1,011)	5.65 (4.49, 7)	1744 (1,425, 2098)	5.43 (4.43, 6.53)	0.01	19,763 (15,785, 24,530)	136.9 (109.35, 169.93)	39,443 (32,034, 47,768)	122.71 (99.66, 148.61)	−0.23***
					(−0.09 to 0.11)					(−0.34 to −0.12)
Eastern Sub-Saharan Africa	1,001 (784, 1,216)	8.23 (6.45, 9.99)	1,688 (1,343, 2,118)	6.24 (4.97, 7.84)	−1.11***	24,740 (19,357, 30,296)	203.36 (159.11, 249.03)	39,284 (31,083, 49,699)	145.29 (114.96, 183.81)	−1.29***
					(−1.19 to −1.03)					(−1.36 to −1.22)
Central Sub-Saharan Africa	294 (209, 386)	7.83 (5.56, 10.25)	613 (443, 794)	6.8 (4.91, 8.8)	−0.49***	7,393 (5,165, 9,828)	196.6 (137.36, 261.37)	14,726 (10,530, 19,141)	163.2 (116.69, 212.13)	−0.61***
					(−0.63 to −0.34)					(−0.74 to −0.47)
Southern Sub-Saharan Africa	421 (345, 564)	9.51 (7.8, 12.75)	909 (806, 1,023)	9.34 (8.27, 10.51)	−0.26	9,456 (7,770, 12,648)	213.71 (175.61, 285.85)	19,483 (17,196, 22,011)	200.13 (176.64, 226.1)	−0.39*
					(−0.55 to 0.02)					(−0.73 to −0.04)

EAPC, estimated annual percentage changes; CI, confidence interval; SDI, sociodemographic index; UI, uncertainty interval.*p-value < 0.05; **p-value < 0.01; ***p-value < 0.001.

### 3.2 SDI regional level

The period from 1990 to 2021 showed a general decrease in prevalence, incidence, mortality, and DALY rates in the five SDI regions (Figure 1 and Table 1). Notably, the low-middle SDI region showed the most minor reductions in all four measures. In contrast, the high-middle SDI region exhibited significantly more significant reductions in prevalence, mortality, and DALY rates than the other regions. In 2021, the high SDI region demonstrated the highest prevalence case and morbidity rates, with 268,129 (253,063 to 279,715) and 77.72 (73.35 to 81.07) per 100,000, respectively; in High-middle SDI, the incidence rate peaked at 12.15 (10.96 to 13.4) per 100,000. The region exhibiting the highest mortality and DALY rates was identified as low-middle SDI, at 9.9 (8.91 to 11.17) and 239.34 (215.3 to 271) cases for every 100,000 people, correspondingly, as shown in Figure 1 and Table 1. Furthermore, the middle SDI region showed the most significant cases of incidence, deaths, and DALYs, with respective figures of 44,598 (39,812 to 49,987), 29,787 (26,773 to 33,198), and 694,235 (623,988 to 773,960), respectively.

### 3.3 GBD regional levels

**Figure 2:**
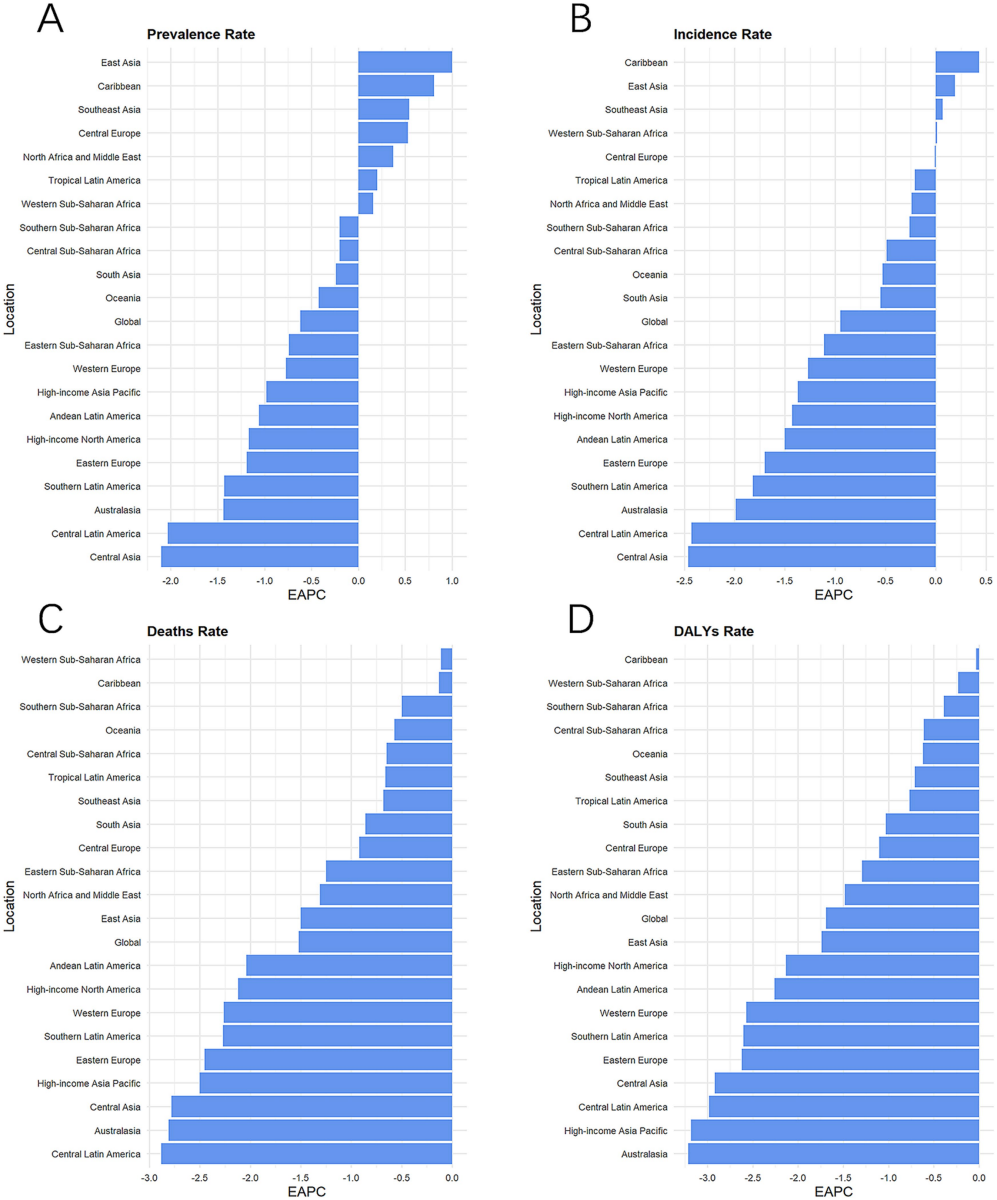
Disease trends in global and 21 regions from 1990 to 2021. The EAPC of prevalence rate **(A)**, incidence rate **(B)**, death rate **(C)**, and DALYs rate **(D)**. DALYs, disability-adjusted life-year; GBD, Global Burden of Disease; EAPC, estimated annual percentage changes.

During the last 32 years, among the 21 regions, mortality and DALY rates dropped, while prevalence and morbidity demonstrated an upward trend in certain regions. Specifically, seven regions exhibited increasing trends in prevalence: East Asia, the Caribbean, Southeast Asia, Central Europe, North Africa and the Middle East, Tropical Latin America, and Western Sub-Saharan Africa (Figure 2 and Table 1). Notably, increases in prevalence were exclusively observed in the Caribbean, East Asia, and Southeast Asia, with EAPCs of 0.43 (0.32 to 0.53), 0.19 (0.04 to 0.34), and 0.07 (0.03 to 0.11), respectively. These regions also demonstrated increasing prevalence trends (Figure 2 and Table 1).

From 1990 through 2021, minor reductions in mortality and DALY rates have been observed in the Caribbean, Western Sub-Saharan Africa, and Southern Sub-Saharan Africa, with the most significant reductions in prevalence, incidence, and mortality demonstrated in Central Asia (Figure 2). The regions with the most considerable DALY rate reductions were Australia, high-income Asia, and Central Pacific Latin America, respectively (Figure 2D). Notably, Central Europe, a region with a high-moderate SDI, had the highest prevalence rate and incidence rate in 2021 at 111 (102.45 to 120.09) and 20.27 (18.55 to 22.09) per 100,000 people, as well as the second highest rate of deaths and DALYs (Figure 2 and Table 1). The number of DALYs reported in South Asia in 2021 was 709,622 (618,985 to 816,799) cases, about one-third of the global number of DALYs.

### 3.4 Country level

**Figure 3:**
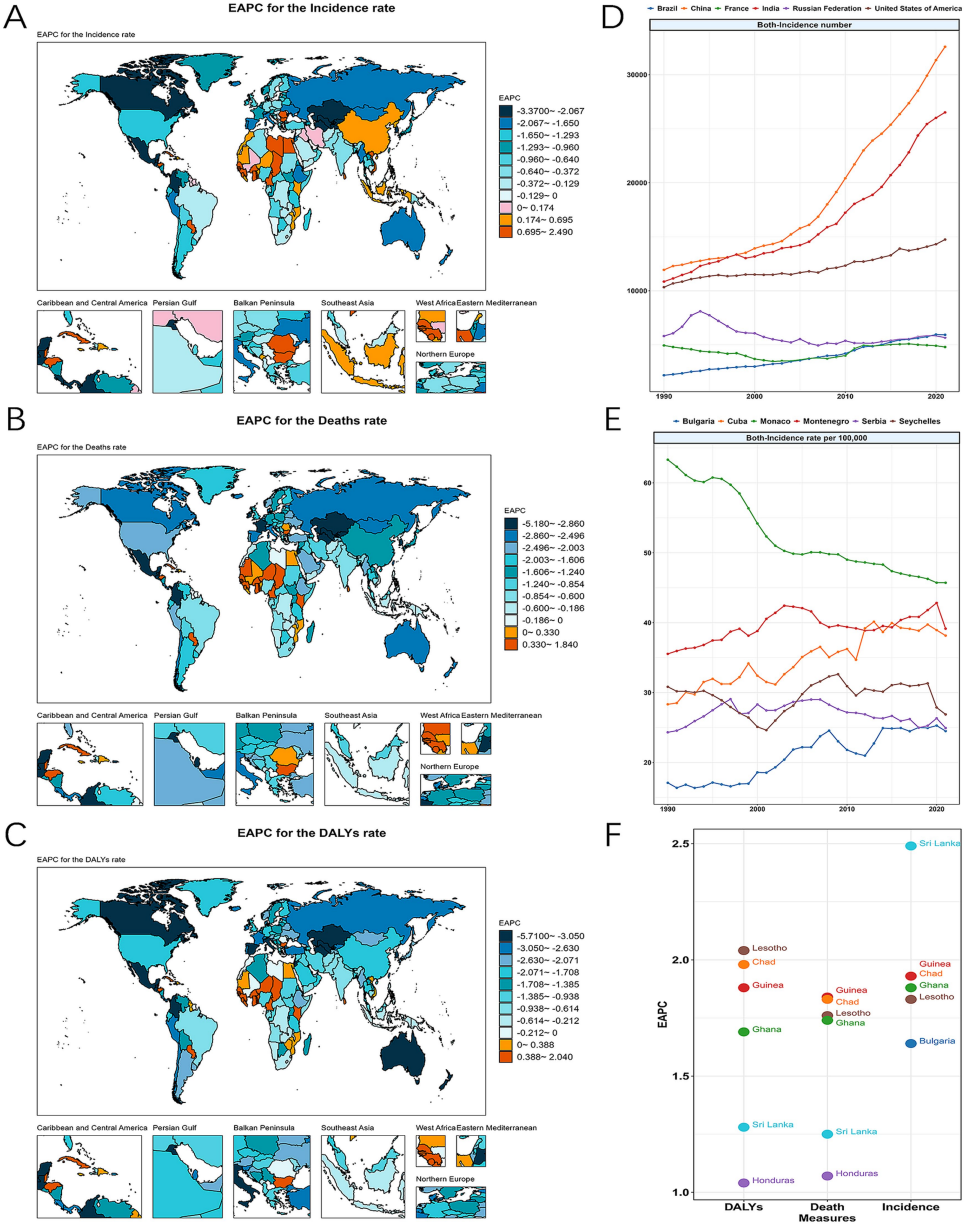
The global burdens of LC in MAOP for both sexes across 204 countries from 1990 to 2021 and the ranks of the leading locations. The EAPC of incidence rate **(A)**, death rate **(B)**, and DALY rate **(C)** in 2021 worldwide and the top six countries. The trend of incidence number **(D)**, incidence rate **(E)** of in the top 6 ranked countries for LC in MAOP from 1990 to 2021. The EAPC of DALY, incidence, and mortality in the top 6 ranked countries **(F)**.

Between 1990 and 2021, the burden of LC in MAOP in most countries declined. However, the proportion of countries with an increasing burden of disease was 24% for incidence rate, 38% for prevalence rate, 14% for mortality rate, and 16% for DALY rate (Figures 3A–C; Supplementary Figure S1; Supplementary Tables S2–S5). The countries with the highest increases in mortality and DALY rates were Guinea and Lesotho, with EAPC of 1.84 (1.64 to 2.05) and 2.04 (1.68 to 2.39), respectively. The top five countries with the increase in the rate of incidence, mortality, and DALY are Guinea, Chad, Lesotho, Ghana, and Sri Lanka (Figure 3F). It is worth noting that Sri Lanka had the highest prevalence and incidence increase, with EAPC of 3.02 (2.59 to 3.47) and 2.49 (2.05 to 2.94), respectively (Figure 3F).

In 2021, China, India, the United States of America, Brazil, the Russian Federation, and France ranked in the top six cases of incidence and prevalence (Figure 3D and Supplementary Tables S2, S3). China and India had the highest cases of incidence, prevalence, mortality, and DALY due to their large populations. In 2021, China’s incidence and DALY case numbers were 32,588 (25,555 to 41,417) and 386,575 (301,814 to 490,830). The Indian numbers for this number are 26,512 (23,106 to 30,559) and 543,682 (472,859 to 627,443). With rates of 45.71 (33.98 to 61.77), 39.14 (30.41 to 51.66), and 38.15 (31.59 to 46.67) per 100,000 population, respectively, Monaco, Montenegro, and Cuba were the leading three nations by incidence rate, which also occupied the top three positions in terms of prevalence rate (Figure 3E and Supplementary Figures S2, S3). Monaco saw a notable decline during these 32 years, while Montenegro and Cuba saw increases (Figure 3F). The top four countries of death rate and DALYs rate were Cuba, Montenegro, Pakistan, and Seychelles (Supplementary Figures S4, S5). That year, India, China, Brazil, Pakistan, the United States of America, and the Russian Federation were the top six nations in mortality and DALY cases. These nations especially need to improve their efforts to prevent and control disease.

### 3.5 Patterns by sex and age group

**Figure 4:**
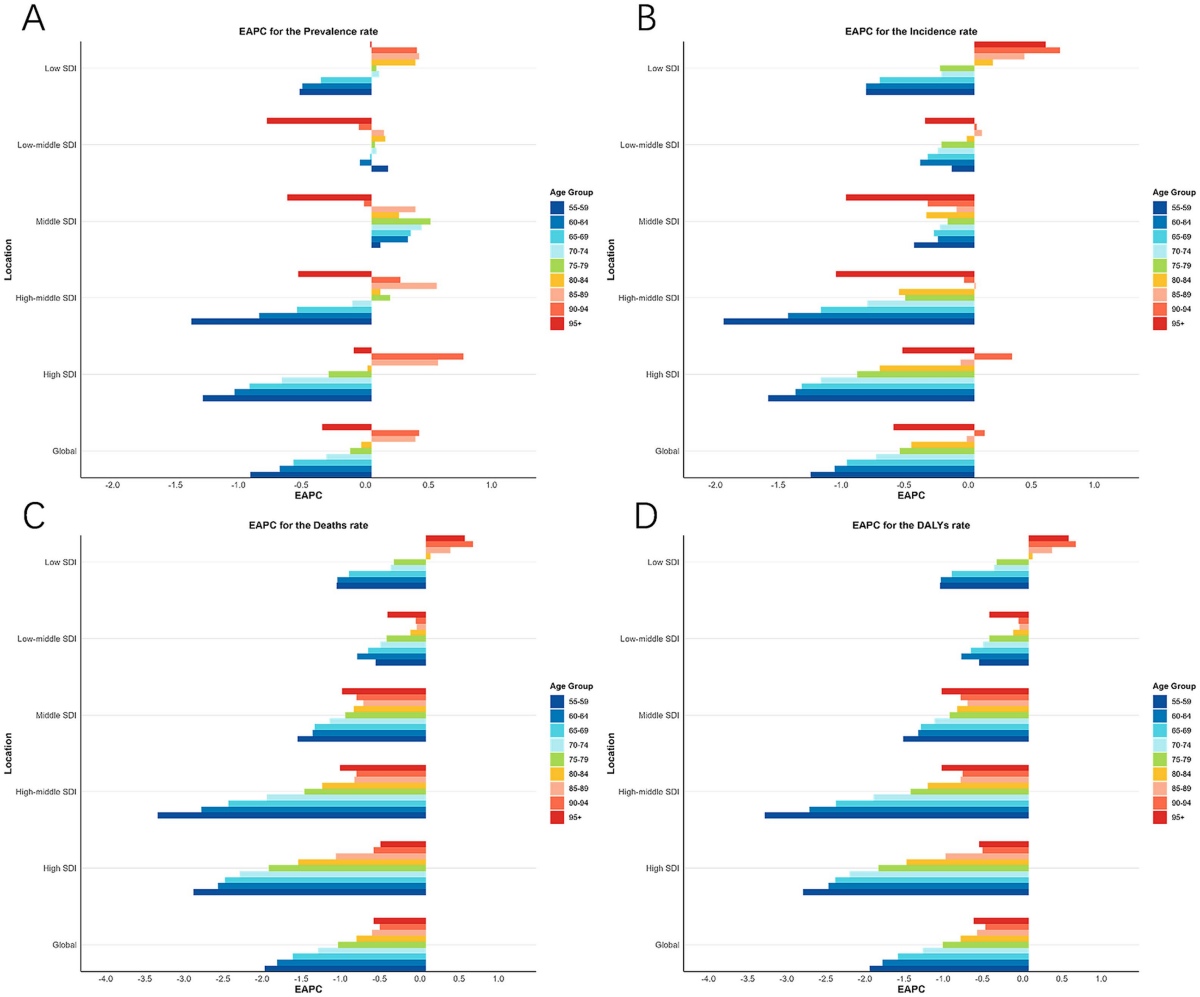
EAPC of prevalence rate **(A)**, incidence rate **(B)**, death rate **(C)**, and DALYs rate **(D)** in 9 age groups in Global and 5 SDI regions from 1990 to 2021. DALYs, disability-adjusted life-year; SDI, socio-demographic index; EAPC, estimated annual percentage changes.

Globally, mortality and DALY rate in the MAOP age groups demonstrated a general tendency toward decline from 1990 through 2021 (Figures 4C,D), while prevalence rates in the 85–89 and 90–94 age groups showed an increase, with the EAPC for these age groups 0.35 (0.3 to 0.4), and 0.38 (0.34 to 0.43), respectively (Figure 4A and Table 2).

**Table 2:** Prevalence cases and rates of LC in MAOP for both sexes in 1990 and 2021, and its temporal trends from 1990 to 2021.

Location and age	1990		2021		1990–2021
	Counts	Rate	Counts	Rate	EAPC
	(95%UI)	pre 100,000 (95%UI)	(95%UI)	pre 100,000 (95%UI)	(95%CI)
Global					
55–59	108,443 (102,828, 114,775)	58.55 (55.52, 61.97)	172,250 (159,345, 186,760)	43.53 (40.27, 47.19)	−0.96*** (−1.05 to −0.88)
60–64	122,221 (116,517, 128,610)	76.1 (72.55, 80.08)	199,399 (187,271, 213,700)	62.3 (58.51, 66.77)	−0.73*** (−0.83 to −0.63)
65–69	102,073 (96,715, 108,100)	82.58 (78.24, 87.45)	197,516 (184,231, 214,407)	71.6 (66.79, 77.73)	−0.62*** (−0.72 to −0.52)
70–74	63,770 (59,625, 67,766)	75.32 (70.43, 80.04)	149,883 (139,409, 161,611)	72.82 (67.73, 78.51)	−0.36*** (−0.45 to −0.27)
75–79	41,553 (38,333, 44,825)	67.5 (62.27, 72.82)	86,774 (80,246, 92,669)	65.8 (60.85, 70.27)	−0.17*** (−0.23 to −0.11)
80–84	19,089 (17,064, 21,280)	53.96 (48.24, 60.15)	46,706 (42,042, 50,324)	53.33 (48, 57.46)	−0.08 (−0.17 to 0.01)
85–89	7,590 (6,737, 8,575)	50.23 (44.58, 56.75)	25,695 (22,242, 27,828)	56.2 (48.65, 60.86)	0.35*** (0.3 to 0.41)
90–94	1737 (1,472, 2,049)	40.54 (34.35, 47.83)	8,217 (7,035, 9,097)	45.93 (39.32, 50.85)	0.38*** (0.34 to 0.43)
95+	237 (165, 345)	23.25 (16.2, 33.91)	1,139 (953, 1,404)	20.91 (17.48, 25.76)	−0.39*** (−0.41 to −0.37)
High SDI					
55–59	37,116 (35,119, 39,141)	87.22 (82.53, 91.98)	40,848 (38,656, 43,052)	56.16 (53.15, 59.19)	−1.34*** (−1.43 to −1.25)
60–64	44,470 (42,629, 46,945)	111.43 (106.82, 117.63)	55,262 (52,587, 58,287)	81.07 (77.14, 85.5)	−1.09*** (−1.17 to −1.02)
65–69	41,605 (39,617, 43,732)	119.06 (113.37, 125.14)	56,506 (52,755, 59,797)	93.21 (87.02, 98.63)	−0.97*** (−1.08 to −0.87)
70–74	26,579 (25,176, 28,027)	106.11 (100.51, 111.89)	49,126 (46,049, 51,771)	92.06 (86.3, 97.02)	−0.71*** (−0.85 to −0.57)
75–79	18,895 (17,445, 20,444)	89.24 (82.39, 96.56)	31,224 (28,736, 33,043)	86.22 (79.35, 91.25)	−0.34*** (−0.49 to −0.18)
80–84	9,058 (8,091, 10,123)	66.45 (59.36, 74.26)	18,028 (15,821, 19,519)	66.84 (58.66, 72.37)	−0.03 (−0.2 to 0.13)
85–89	4,066 (3,584, 4,611)	62.41 (55.02, 70.79)	11,959 (10,258, 13,021)	72.49 (62.18, 78.92)	0.53*** (0.45 to 0.6)
90–94	1,010 (844, 1,193)	47.33 (39.55, 55.93)	4,530 (3,819, 5,021)	59.09 (49.81, 65.5)	0.73*** (0.67 to 0.79)
95+	138 (93, 205)	24.73 (16.65, 36.82)	645 (533, 803)	23.28 (19.24, 28.98)	−0.14*** (−0.19 to −0.09)
High-middle SDI					
55–59	36,640 (34,648, 38,645)	78.81 (74.53, 83.13)	46,691 (41,622, 51,872)	51.92 (46.28, 57.68)	−1.43*** (−1.57 to −1.28)
60–64	41,222 (39,057, 43,789)	95.76 (90.73, 101.72)	56,116 (50,327, 62,331)	76.94 (69, 85.46)	−0.89*** (−1.02 to −0.77)
65–69	29,860 (27,937, 31,845)	94.72 (88.62, 101.02)	55,549 (49,565, 62,186)	83.65 (74.64, 93.65)	−0.59*** (−0.7 to −0.48)
70–74	16,818 (15,636, 18,125)	81.44 (75.72, 87.77)	39,567 (35,246, 44,299)	81 (72.15, 90.69)	−0.15*** (−0.23 to −0.08)
75–79	11,617 (10,659, 12,636)	69.79 (64.04, 75.92)	21,544 (19,258, 23,926)	72.13 (64.48, 80.11)	0.15*** (0.1 to 0.21)
80–84	5,033 (4,460, 5,694)	54.07 (47.91, 61.17)	11,883 (10,615, 13,020)	54.43 (48.62, 59.64)	0.07 (−0.01 to 0.15)
85–89	1782 (1,553, 2040)	47.66 (41.53, 54.55)	6,639 (5,809, 7,295)	58.35 (51.06, 64.12)	0.52*** (0.45 to 0.6)
90–94	369 (310, 442)	39.64 (33.33, 47.53)	1909 (1,667, 2,123)	43.68 (38.13, 48.57)	0.23*** (0.16 to 0.31)
95+	50 (36, 71)	26.42 (18.88, 37.81)	256 (216, 311)	23.15 (19.53, 28.14)	−0.58*** (−0.69 to −0.47)
Middle SDI					
55–59	18,021 (16,182, 19,793)	33.9 (30.44, 37.23)	48,203 (42,309, 54,707)	34.94 (30.67, 39.66)	0.07 (−0.07 to 0.21)
60–64	18,830 (17,124, 20,568)	43.96 (39.98, 48.02)	50,452 (44,681, 56,728)	49.57 (43.9, 55.73)	0.29*** (0.17 to 0.42)
65–69	16,131 (14,539, 17,739)	50.62 (45.62, 55.66)	51,507 (45,283, 58,945)	57.78 (50.8, 66.13)	0.31*** (0.19 to 0.42)
70–74	11,247 (10,037, 12,266)	51.46 (45.92, 56.12)	37,009 (32,713, 41,884)	60.05 (53.08, 67.96)	0.4*** (0.32 to 0.48)
75–79	6,202 (5,558, 6,718)	46.39 (41.57, 50.24)	21,014 (18,826, 23,540)	53.63 (48.05, 60.08)	0.47*** (0.4 to 0.55)
80–84	2,756 (2,428, 3,010)	39.77 (35.04, 43.43)	10,406 (9,224, 11,464)	43.68 (38.72, 48.13)	0.22*** (0.17 to 0.27)
85–89	1,032 (920, 1,145)	37.83 (33.71, 41.98)	4,877 (4,223, 5,393)	42.53 (36.82, 47.03)	0.35*** (0.29 to 0.41)
90–94	204 (176, 237)	30.83 (26.63, 35.81)	1,221 (1,048, 1,365)	31.66 (27.19, 35.41)	−0.06 (−0.14 to 0.02)
95+	25 (19, 34)	17.18 (12.97, 23.44)	157 (131, 193)	15.12 (12.6, 18.58)	−0.67*** (−0.76 to −0.58)
Low-middle SDI					
55–59	12,572 (10,609, 15,018)	40.43 (34.11, 48.29)	28,894 (25,236, 33,016)	41.6 (36.33, 47.53)	0.13 (−0.03 to 0.28)
60–64	13,377 (11,467, 15,642)	52.86 (45.31, 61.8)	29,462 (26,666, 33,115)	51.54 (46.65, 57.93)	−0.09 (−0.2 to 0.02)
65–69	10,870 (9,244, 12,774)	59.82 (50.88, 70.3)	26,780 (23,529, 30,484)	60.37 (53.04, 68.72)	−0.01 (−0.13 to 0.11)
70–74	6,890 (5,834, 8,005)	56.07 (47.48, 65.15)	19,039 (16,973, 21,604)	60.54 (53.97, 68.7)	0.04 (−0.09 to 0.17)
75–79	3,681 (3,116, 4,284)	48.57 (41.11, 56.52)	10,223 (9,138, 11,679)	50.91 (45.51, 58.17)	0.03 (−0.05 to 0.12)
80–84	1769 (1,464, 2,105)	42.51 (35.16, 50.56)	5,109 (4,536, 5,840)	44.43 (39.44, 50.78)	0.11* (0.02 to 0.19)
85–89	575 (485, 688)	34.77 (29.34, 41.62)	1796 (1,591, 2,023)	35.93 (31.85, 40.49)	0.1 (0 to 0.2)
90–94	128 (106, 154)	29 (23.96, 34.9)	461 (403, 524)	28.48 (24.91, 32.36)	−0.1 (−0.21 to 0.02)
95+	20 (15, 29)	19.99 (14.77, 28.47)	70 (58, 85)	15.44 (12.9, 18.71)	−0.83*** (−0.94 to −0.72)
Low SDI					
55–59	3,924 (3,117, 4,895)	33.57 (26.67, 41.88)	7,374 (6,163, 8,609)	29.11 (24.33, 33.99)	−0.57*** (−0.71 to −0.44)
60–64	4,145 (3,297, 5,090)	44.41 (35.33, 54.53)	7,818 (6,639, 9,141)	39.65 (33.67, 46.36)	−0.55*** (−0.67 to −0.43)
65–69	3,468 (2,729, 4,321)	49.88 (39.25, 62.15)	6,910 (5,876, 8,042)	45.84 (38.98, 53.34)	−0.4*** (−0.51 to −0.29)
70–74	2,163 (1769, 2,643)	45.76 (37.42, 55.9)	4,967 (4,287, 5,821)	47.89 (41.33, 56.13)	0.06 (−0.03 to 0.15)
75–79	1,103 (880, 1,330)	40.66 (32.44, 49.01)	2,673 (2,326, 3,044)	41.62 (36.22, 47.4)	0.04 (−0.02 to 0.1)
80–84	446 (349, 533)	34.34 (26.87, 41.07)	1,228 (1,043, 1,425)	36.39 (30.93, 42.25)	0.35*** (0.2 to 0.5)
85–89	124 (101, 150)	27.19 (22.24, 32.93)	399 (344, 459)	29.9 (25.8, 34.38)	0.38** (0.18 to 0.59)
90–94	25 (20, 30)	21.71 (17.72, 26.68)	88 (76, 99)	24.47 (21.31, 27.71)	0.36*** (0.17 to 0.54)
95+	4 (3, 5)	14.74 (10.73, 20.97)	10 (9, 13)	13.56 (11.25, 16.69)	−0.01 (−0.13 to 0.12)

EAPC, estimated annual percentage changes; CI, confidence interval; SDI, sociodemographic index; UI, uncertainty interval. *p-value < 0.05; **p-value < 0.01; ***p-value < 0.001.

**Figure 5:**
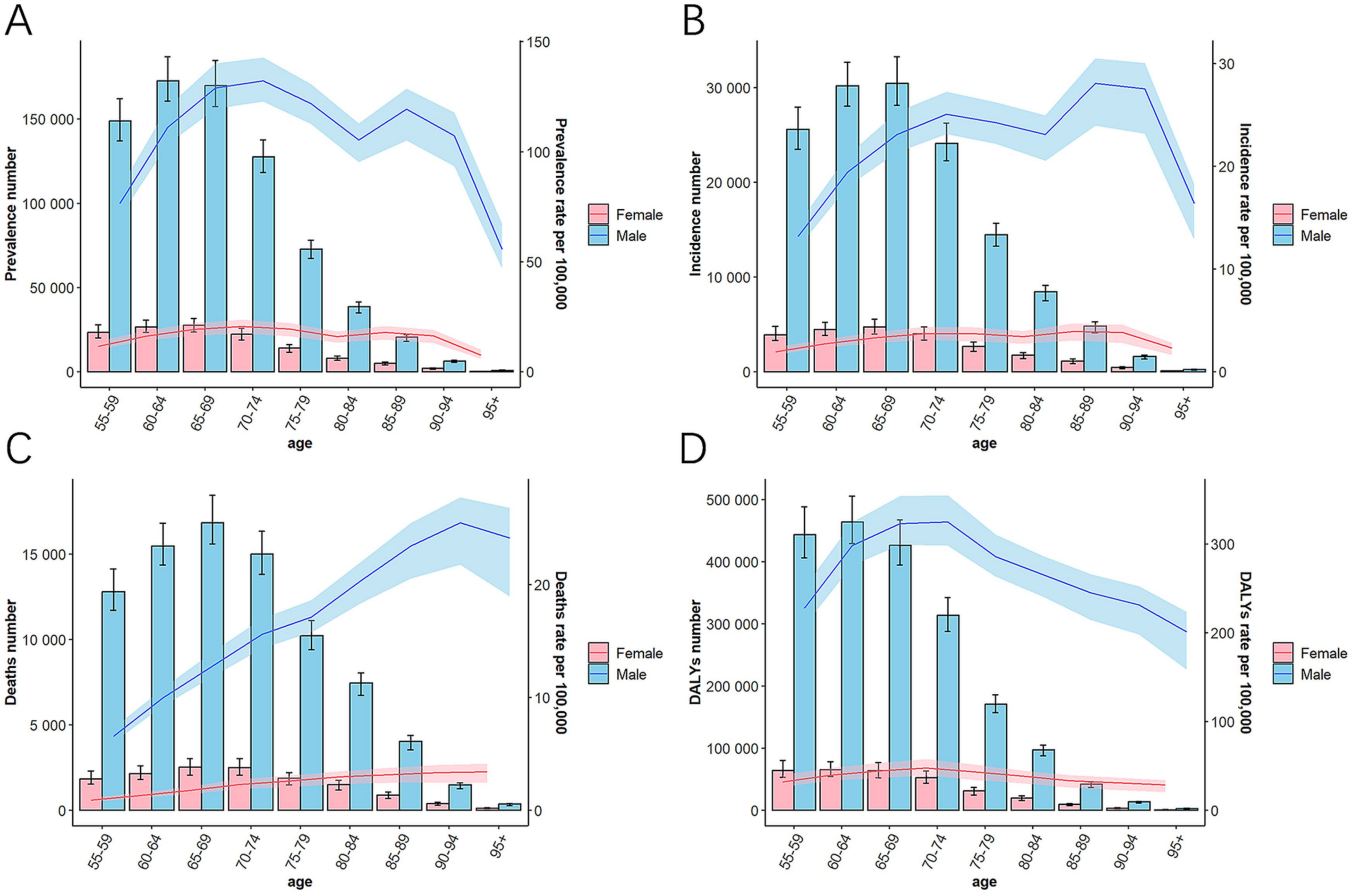
The case and rate of prevalence **(A)**, incidence **(B)**, death **(C)**, and DALYs **(D)** in 9 age groups globally in 2021. DALYs, disability-adjusted life-year; SDI, socio-demographic index.

Over the past 32 years, global reductions in the rate of prevalence, morbidity, mortality, and DALY were slightly better for men than for women (Table 1 and Figure 1). However, in 2021, globally, the rate of these indicators was considerably more severe for male than for female in different age groups and across SDI regions (Figures 1, 4 and Supplementary Figure S6). Specifically, the case of incidence and mortality in men worldwide were 140,040 (130,074 to 151,216) and 83,699 (77,817 to 90,455), respectively; the DALYs rate was 282.12 (261.85, 305.98) per 100,000, while the morbidity rate in women was 23,276 (20,094 to 26,886) (Table 1). The number and rate of prevalence, incidence, and DALYs were superior in males among MAOP before age 75; incidence and prevalence rates increased again between ages 80 and 94 years, with a peak prevalence rate of 28.05 (23.93, 30.47) per 100,000 persons in men aged 85 to 90 years. The prevalence rate and DALYs rates peaked in males aged 70–74 years at 132.21 (122.68, 142.76) per 100,000 population and 47.78 (39.55, 57.81) for every 100,000 people, while a secondary peak was observed in the prevalence rate of males aged 85–89 years at 119.37 (105.01, 128.56) per 100,000 population. The peak number of DALY was in the 60–64 age group, with 463,638 (429,177 to 504,950). The mortality rate exhibited an age-dependent increase, with the maximum case of deaths occurring aged 65–69 years, amounting to 16,841 (15,597 to 18,459) (Figure 5; Table 2; Supplementary Tables S6–S8).

Over the past 32 years, an age-related worsening trend has been observed in the global and five SDI regional burden of disease indicators, including prevalence, incidence, mortality, and DALY rates (Figure 4; Table 2; Supplementary Tables S6–S9). Mortality and DALY rates for the MAOP showed a decreasing trend in other regions, except for the 80+ age group in the Low SDI region (Figures 4C,D). However, regarding the incidence rate, increasing trends were observed among the following age groups: individuals aged 80+ years in low SDI regions, 85–94 years in low-middle SDI regions, 80–85 years in high-middle SDI regions, and 90–94 years in high SDI regions, while the remaining age groups show decreasing trends (Figure 4B and Supplementary Table S7). Regarding prevalence rate, several regions, and age groups still presented increasing trends, with the high-middle SDI region showing the best-improving trends in the rate of incidence and mortality and the low SDI region demonstrating the worst (Figure 4). The 55–59 age group showed the best improvement in prevalence, incidence, mortality, and DALY. It is noteworthy that the prevalence and incidence of females in high SDI regions remained persistently elevated relative to other SDI regions (Table 1).

### 3.6 Indicators correlation with SDI

Across most regions, SDI was positively correlated with prevalence and incidence rate, with correlation coefficients (*R*-values) of 0.604 and 0.038, respectively (Supplementary Figures S7, S8). Conversely, the rate of DALY and mortality exhibited an overall negative correlation with SDI, with correlation coefficients (*R*-values) of −0.122 and −0.154, respectively (Supplementary Figures S9, 10). Initially, the prevalence and incidence rates increased but decreased as the SDI level continued to rise. For mortality rate and DALYs rate, these indicators demonstrated slight fluctuations with the SDI until it reached 0.7, after which they began to decline. For instance, high SDI regions (e.g., Western Europe) had higher prevalence and morbidity rates, while their mortality and DALY rates are relatively low.

### 3.7 Risk factor analysis

Based on GBD 2021 data, our study evaluated the secondary-level contributable risk factors linked to LC in MAOP. Three risk factors were identified for laryngeal cancer mortality and DALY, including alcohol use, tobacco use, and occupational risk. Notably, tobacco was the leading risk factor, contributing substantially to laryngeal cancer mortality and DALYs in middle-aged and older adults, followed by alcohol consumption, and occupational risk factors were relatively minor. In 2021, the global numbers of deaths associated with alcohol use, tobacco use, and occupational hazards were 11,840 (6,542 to 17,002), 66,667 (59,522 to 73,780), and 5,885 (3,674 to 8,534), respectively, with corresponding death rates of 0.8 (0.44 to 1.14), 4.49 (4.01 to 4.97), and 0.4 (0.25 to 0.57) per 100,000 people. These factors contributed to DALY rates of 19.6 (10.79 to 27.66), 106.39 (95.51 to 117.35), and 9.06 (5.64 to 13.37) per 100,000 people, respectively.

**Figure 6:**
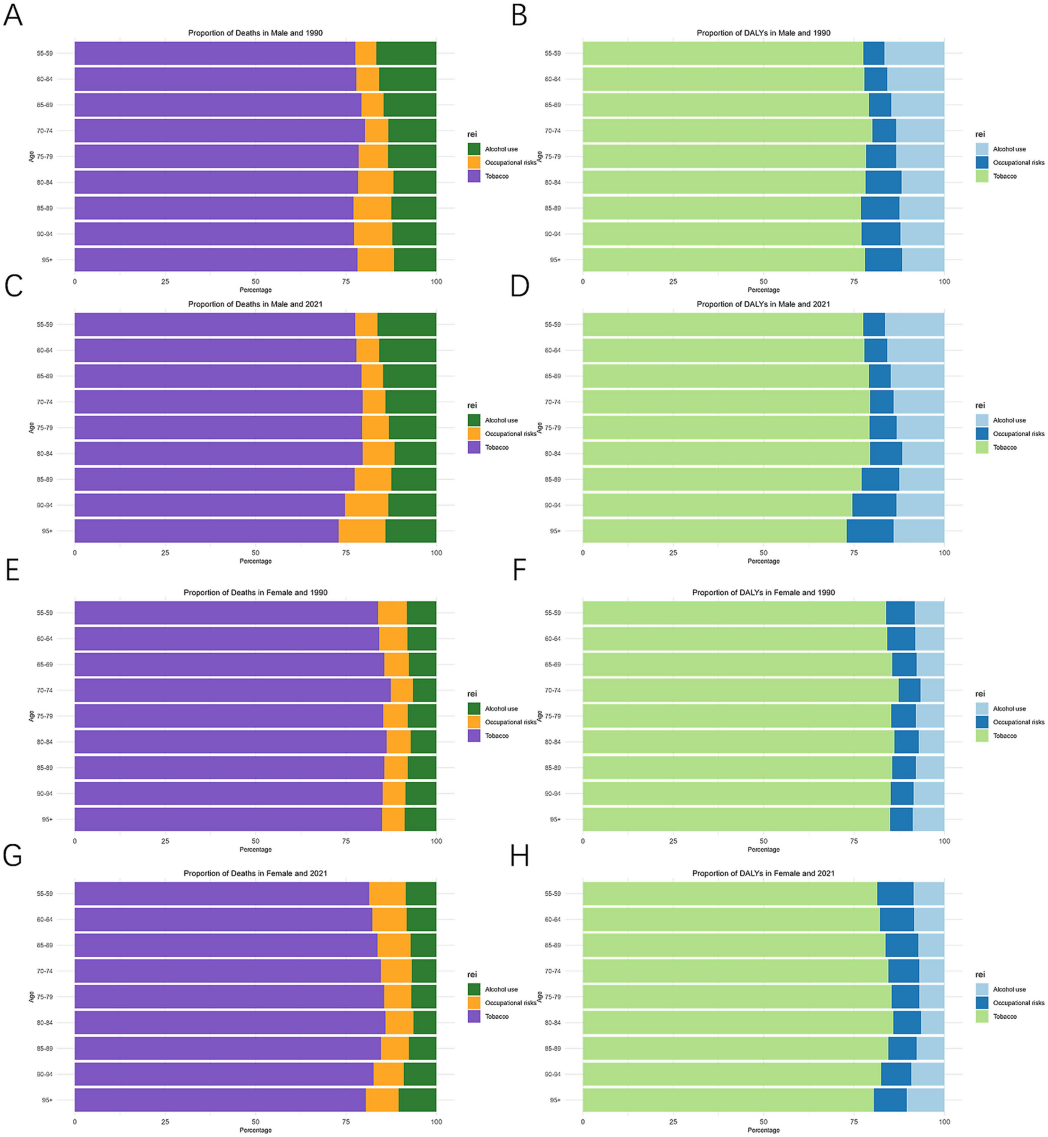
Proportion of death and DALYs by age and gender attributable to risk factors in 1990 and 2021. Death **(A)** and DALYs **(B)** in males and 1990, Death **(C)** and DALYs **(D)** in males and 2021, Death **(E)** and DALYs **(F)** in females and 1990, Death **(G)** and DALYs **(H)** in female and 2021. DALYs, disability-adjusted life-year.

**Figure 7:**
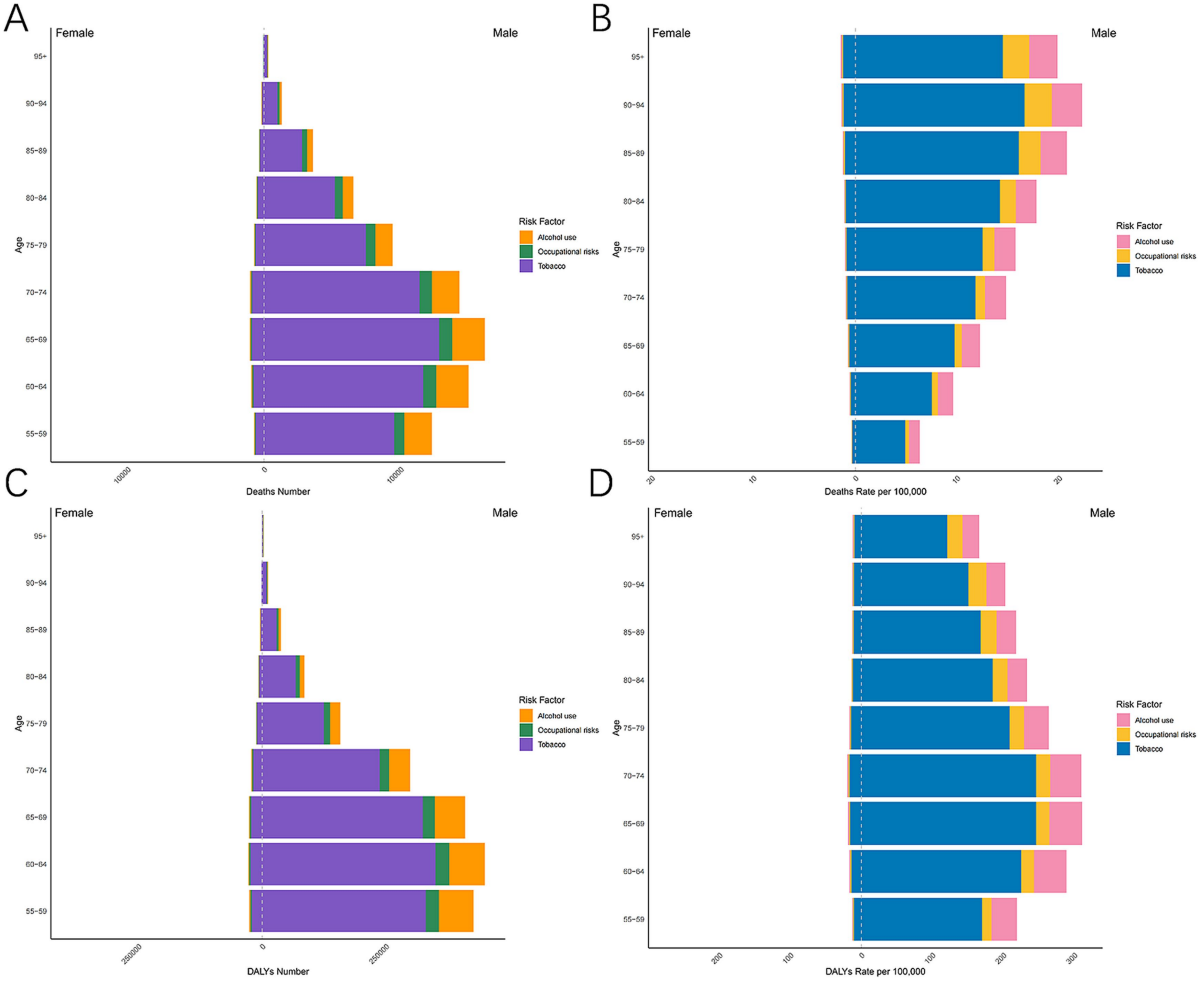
The case and rate of mortality and DALY associated with alcohol use, tobacco use, and occupational risk in 9 age groups globally in 2021. Death number **(A)**, Death rate **(B)**, DALY number **(C)**, DALYs rate **(D)**. DALYs, Disability-adjusted life-year.

**Figure 8:**
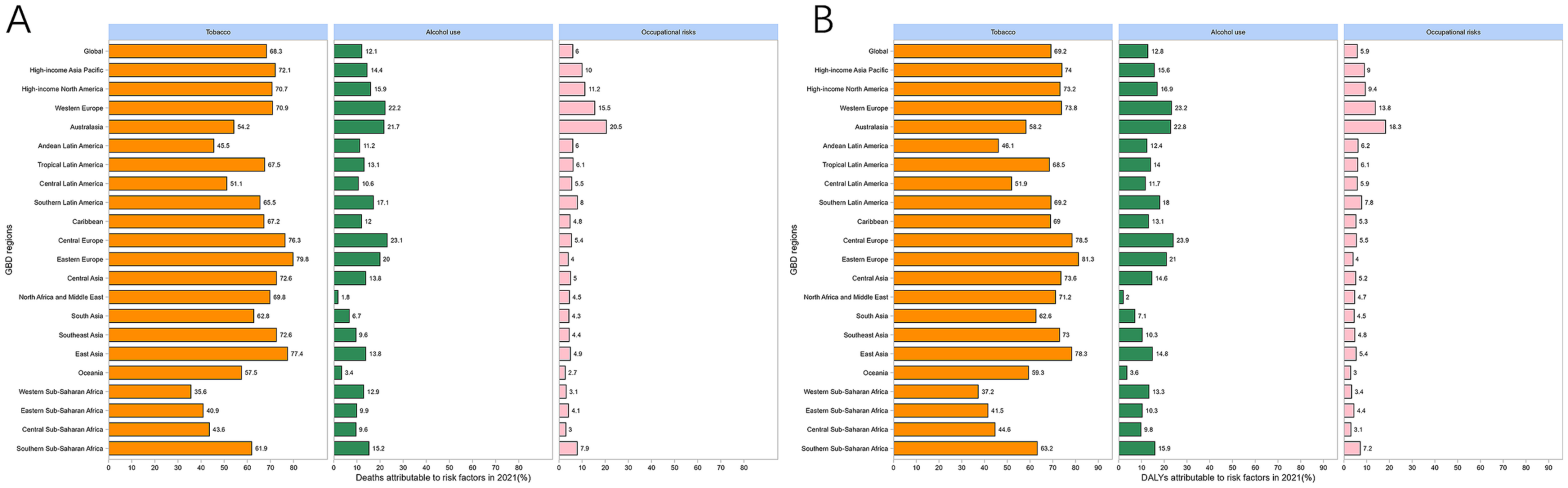
Proportion of laryngeal cancer death **(A)** and DALYs **(B)** attributable to alcohol, tobacco, and occupational risk among 21 GBD regions. DALYs, disability-adjusted life-year.

Mortality and DALY rates associated with risk factors demonstrated systematic gender/age variations in LC in MAOP associated with risk factors. Men faced a much greater burden of disease related to tobacco and alcohol use than women, illustrating gender disparities (Figure 6). The number of DALYs and mortality related to alcohol use, tobacco use, and occupational risks increased initially and then decreased with age (Figures 7A,C). Conversely, mortality rates exhibited an increasing trend with age, peaking at 90–94 years for males (Figure 7B). DALY rates related to the three risk factors increased with age up to 74 years, after which a decreasing trend was observed (Figure 7D). Compared with 1990, the 2021 data revealed a decline in the proportion of tobacco-related deaths and DALYs among the male population aged over 90, while the proportion attributable to alcohol consumption and occupational risks increased (Figures 6A–D). A slight uptick was observed in the percentage of deaths and DALYs among females due to LC from occupational factors (Figures 6E–H). The three regions with the highest burden of DALY and deaths due to smoking were Eastern Europe, Central Europe, and East Asia (Figure 8).

**Figure 9:**
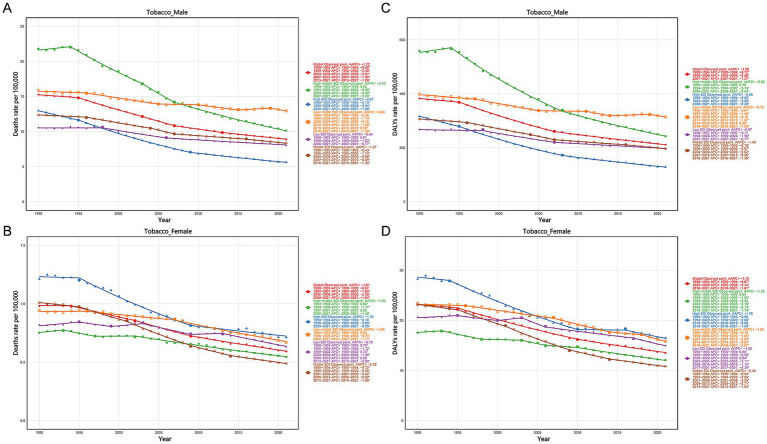
Temporal trends of mortality and DALY rates attributed to tobacco of LC in MAOP globally and in five SDI regions. Death rate attributable to tobacco in male **(A)** and female **(B)**. DALYs rate attributable to tobacco in male **(C)** and female **(D)**. MAOP, middle-aged and older populations; LC, laryngeal cancer; DALYs, disability-adjusted life-year.

Since the early 1990s, the mortality and DALY rates of LC in MAOP attributed to tobacco, alcohol use, and occupational factors demonstrated a downward trend globally and across five SDI regions, yet regional disparities persist. Males in the high SDI region showed the most remarkable drop in tobacco-related mortality and DALY rates in MAOP (Figures 9A,C). Conversely, the low-middle SDI region saw the least drop among males and became the region with the most excellent tobacco-related mortality rate among men in 2021. Among females, the middle SDI region showed t the most marked reduction, while the low SDI region experienced the least reduction (Figures 9B,D). Notably, although the burden of smoking-attributable LC in MAOP was lowest in males in the high SDI region, the high SDI regions consistently had higher rates of smoking-related mortality and DALY rate in females.

Regarding the mortality rate and DALY of LC in MAOP caused by alcohol consumption, the high middle SDI region had consistently had the highest male alcohol-related mortality rate and DALY rate over the previous 32 years, despite showing the most significant decline in both male and female populations (Supplementary Figure S11). The mortality and DALY rate of alcohol-related LC in MAOP were increasing in both male and female populations in the low-middle SDI and Low SDI regions. Throughout the whole study period, females in high SDI areas had consistently increased alcohol-related death rates and DALYs for LC in MAOP (Supplementary Figures S11B,D).

Both male and female groups revealed a rising tendency in the low-middle SDI area in the occupational factors related to laryngeal cancer mortality and DALY rate, with the male group experiencing the most significant decrease in the high-middle SDI region and the female group in the high SDI region (Supplementary Figure S12). Therefore, it is essential to strengthen the prevention and control of smoking and alcohol consumption in low-middle SDI areas. At the same time, women should strengthen these measures in low SDI and high SDI areas.

## 4 Discussion

Based on the GBD database, this study provided a detailed analysis of the epidemiological patterns of LC in MAOP from 1990 to 2021. It explored the burden of disease in different age groups. The study’s findings indicated that while the overall prevalence, incidence, mortality, and DALY of LC in MAOP decreased globally, the burden of disease indicators increased in specific regions and age-specific groups. Tobacco was notably the primary risk factor, heavily influencing laryngeal cancer mortality and DALYs in MAOP. Moreover, the forecasted global disease burden for laryngeal cancer is expected to continue following the same trends that were observed from 1990 to 2021.

The non-linear correlation between SDI and health burden indicators carried significant policy ramifications. The present study identified a positive relationship between the prevalence of the condition and the SDI, with high SDI regions exhibiting a distinctive pattern characterized by a “high prevalence rate-low mortality rate.” For example, despite higher prevalence and morbidity, Western Europe and North America had significantly lower mortality and DALY rates than low and medium SDI regions. This occurrence might be related to higher alcohol and tobacco use per person (14). At the same time, these developed countries have better early detection systems (e.g., widespread laryngoscopy technology) (15) and targeted therapies (e.g., use of immunotherapy) (16), as well as better disease registration systems (17) and longer survival, which together improve survival and prolong the time patients live with the disease. In contrast, the negatively correlated relationship between mortality and SDI highlights the role of medical technology in improving prognosis. The low SDIs (e.g., sub-Saharan Africa) are limited by inadequate medical resources, with fewer than 50% of individuals diagnosed with cancer achieving survival rates beyond 3 years post-diagnosis (18). Its persistent barriers to cancer treatment, including surgery, radiotherapy, and chemotherapy, may contribute to the observed low cancer survival rates in sub-Saharan Africa. Prioritizing the strengthening of surgical care systems, expanding oncology-specific surgical education programs, and implementing multidisciplinary diagnostic support should constitute critical objectives for healthcare development in low SDI regions.

Central Europe, a high-middle SDI region, was a “hotspot” for the laryngeal cancer burden, with the highest prevalence and incidence rates in 2021. Additionally, it had the second-highest mortality and DALY rates, with a substantial proportion of DALYs and deaths attributed to smoking. Factors contributing to this include differences in unhealthy behaviors, including smoking and drinking (19), along with challenges and inequalities in cancer screening, diagnosis, treatment, and care (20). The Caribbean is also a country with a high burden of laryngeal cancer. Its incidence and prevalence rate were on an upward trend, and the rates of death and DALYs decreased the least. Resources for cancer control are limited due to the geographic distribution of islands in the Caribbean, lack of health system resources, and increased vulnerability to climatic hazards (21), and suboptimal cancer surveillance systems. A key contributor to the Caribbean’s high mortality is the prevalence of late-stage disease at diagnosis, resulting from the factors outlined earlier. These make cancer care in the Caribbean extremely challenging (22).

Epidemiological characterization of gender, age, and risk factors in middle-aged and older laryngeal cancer patients reveals complex interactions between biological differences, environmental exposures, and social behavior patterns. The findings of this study suggest that tobacco use plays a substantial role in the development of laryngeal carcinogenesis. The observed correlation appears mediated through nicotine’s regulatory effects on neoplastic cell dissemination dynamics in head and neck malignancies. This effect is associated with nicotine-altering CHRNA5 gene expression, which accelerates cancer cell metastasis (23). Statistical analyses demonstrated a significant correlation between smoking and the propensity for developing head and neck tumors, inclusive of a dose–response relationship with smoking intensity and longevity.

In instances where tobacco use is non-existent, the connection between alcohol intake and head and neck cancer is relatively modest. This association is evident primarily in the circumstances involving high-dose alcohol consumption and the occurrence of LC (24). This is consistent with the burden of smoking and alcohol in our study. Notably, individuals who chew tobacco exhibit an increased risk of laryngeal cancer (25). Especially in India and Sri Lanka, due to their unique habit of chewing tobacco, there is an urgent need to carry out targeted etiological research (26). The daily quantity of smoking is a significant indicator of disease risk. Middle-aged and older men bear a substantially greater share of smoking-related head and neck cancer cases compared to women. This phenomenon is closely related to gender differences in tobacco consumption. In 2019, the number of men aged 30 or over who smoke 20 or more cigarettes per day reached 231 million (32.3%), compared to 27.1 million women (18.5%) (27). In terms of the geographical distribution of smoking, studies have shown a significant increase in smoking prevalence in North Africa, the Middle East, and sub-Saharan Africa since 1990, with China accounting for over a third of global tobacco demand. In 2019, countries with the highest per capita tobacco consumption were primarily located in Europe, such as Montenegro, where the health burden of head and neck cancer among middle-aged and older individuals is relatively high. In low-income countries, over 2.8 billion people remain unprotected by smoke-free environment laws, and strict restrictions on tobacco marketing cover only 18% of the global population (28). Moreover, studies indicate that adult smoking rates are higher in high-income groups of wealthier countries, while the average adult smoking rate in middle-low-income countries is nearly twice that of low-income countries (LICs) (23% vs. 12%, respectively) (29).

Studies show a correlation between the age of the onset of tobacco-related deaths and the economic level of the country. Death caused by tobacco among the older population was higher in LMICs and upper-middle-income countries (UMICs), with proportions of 56 and 51%, respectively, as compared to 74% in LICs (29). The cost of smoking-related mortality as a proportion of the total socioeconomic cost of tobacco use was 64 and 83% in Chad and Sri Lanka, respectively, highlighting the severity of the tobacco-related burden in these two countries (29). Therefore, there is an urgent need to strengthen tobacco control measures in these countries. The study found that the proportion of smoking-attributable deaths increased with age, peaking at 60–64 (22.0% of smoking-attributable deaths), and then declined in older age groups (27), which is generally consistent with our findings. In the MAOP, the relatively long duration of smoking exposure, coupled with immune senescence, i.e., immune dysfunction that occurs with age in this population, affects tumor growth and the effectiveness of anti-tumor immune reactivity in older adults (30). Alcohol, a recognized risk factor for laryngeal cancers, has a heightened risk associated with the potency and frequency of alcohol consumption. In contrast, the effect of the duration of alcohol consumption is more complex. Ethanol may contribute to cancer development, including alterations or damage to DNA, proteins, and lipids acetaldehyde (7). In addition, specific industrial exposures [including asbestos (31) and benzene] have been associated with LC development. People working in specific industries, such as construction, metals, textiles, ceramics, logging, and food, are more likely to develop laryngeal cancer (32). This suggests the need to strengthen occupational health and safety legislation 2021. The increase in DALYs and deaths due to occupational exposures in women reflects the inadequate protection of female workers in manufacturing industries. In addition to these etiologies, there is evidence that infection with high-risk HPV, particularly HPV16, is a risk factor for LC (8).

Significant variations in the burden of LC worldwide require implementing differentiated control measures based on epidemiological characteristics and development status. It is recommended to actively implement a coordinated strategy for the prevention and control of laryngeal cancer at three levels: primary prevention should focus on tobacco and alcohol control and strengthen relevant legislation, especially for low-income, lower-middle-income and high-risk countries, and occupational safety and health; secondary prevention should promote low-cost screening technology in high-risk areas; and tertiary prevention should optimize treatment protocols for older adult patients. Capacity development for cancer control in LICs should be strengthened (5). The availability of surgical infrastructure, perioperative care, surgical expertise, team building, and comprehensive services should be improved (33).

For high-risk countries, tiered intervention strategies should be implemented, especially in low SDI nations (e.g., Chad), Central Europe and low-middle SDI, where primary prevention should be prioritized. Increasing taxes on tobacco products is the most cost-effective and valid intervention (29). Actively promote HPV vaccination; widespread vaccination has a possibility for a reduction in the incidence of HPV-positive LC (1). Improve the protection of occupationally exposed people, especially in Southeast Asia and low-middle SDI, where wearing N95 masks and laryngeal protection devices should be mandatory. Meanwhile, early detection of laryngeal cancer by peripheral blood exosome extraction improves the diagnostic capacity at the grassroots level (34); a range of advanced imaging techniques (e.g., multimodal optics, micro endoscopy, hyperspectral and infrared thermography) has relatively low-cost and non-invasive characteristics that can be used for rapid diagnosis (35). To improve precision screening and early intervention in populous countries, e.g., China, India, and other populous countries should implement 2-year laryngoscopic screening for high-risk men (≥40 years old, ≥20 pack-years of smoking), particularly in individuals under 75 and over 85 years of age.

Strengthening partnerships between Europe and developing nations will improve the research potential of these regions that are undergoing development (36). Integrating artificial intelligence, including machine learning, deep learning, and robotic surgery, into the overall management of laryngeal cancer can improve the quality of medical care (37). For older adult patients with early-stage disease, minimally invasive surgical or radiation treatments, such as transoral laser microsurgery, offer the potential for organ preservation (9). This revision underscores the significance of the multidisciplinary team (MDT), particularly for individuals with locally advanced cancer. In such cases, organ-sparing surgical procedures, Chemoradiotherapy, or radiotherapy alone can provide the possibility of laryngeal preservation while maintaining overall survival rates (38).

This study was limited by the retrospective characterization of Global Burden of Disease (GBD) data, with incomplete registration of deaths in some countries, particularly in areas with a low SDI. In addition, risk factors like tobacco and alcohol were not further delineated for measurement and stratification, and the etiologic role of HPV in LC was not separately assessed, potentially underestimating the impact of biological factors on epidemiologic patterns. In addition, laryngeal cancers were not examined by subtyping, e.g., glottic and subglottic types. In the future, there is a need to deepen molecular epidemiological studies of laryngeal cancer by combining multi-omics data (e.g., genomic and environmental exposure groups) and to develop region-specific risk prediction models to optimize screening strategies.

## 5 Conclusion

Globally, the burden of LC in MAOP experienced a consistent decline since 1990; however, the disease exhibits significant SDI stratification and substantial age and geographic variations. The disease burden is higher in low and low-middle SDI regions. Prevention and control strategies should be the primary focus before 75 and after 85 years old. The main risk factor for LC related deaths and DALY in MAOP is tobacco exposure, particularly in low-middle SDI regions where the burden is significant. Prevention and control of smoking and alcohol consumption in low SDI and high SDI areas should be strengthened among women. To effectively address the reduction of the global burden of LC in MAOP, it is imperative to comprehensively consider sociodemographic indices, geographic differences, gender disparities, age and risk factors, and the implementation of tailored intervention measures.

## Data Availability

Publicly available datasets were analyzed in this study. This data can be found at: https://ghdx.healthdata.org/gbd-2021.

## Footnotes

^1^ https://vizhub.healthdata.org/gbd-results/
