# Correction: Global, regional, and national burden of laryngeal cancer in middle-aged and older adults from 1990 to 2021: an analysis of age and sex differences and attributable risk factors

**DOI:** 10.3389/fpubh.2025.1641852

**Published:** 2025-06-18

**Authors:** Hongxia Xiao, Hui Wang, Hui Zhang, Shanying Wu, Bo Yu

**Affiliations:** ^1^Department of Otolaryngology, Linyi Central Hospital, Linyi, China; ^2^Department of Otolaryngology, Head and Neck Surgery, The Second Hospital of Weihai City, Weihai, China; ^3^Department of Otolaryngology, Head and Neck Surgery, The Second Hospital of Dalian Medical University, Dalian, China

**Keywords:** laryngeal cancer, global burden of disease, middle-aged, older adult, risk factors

An incorrect **Funding** statement was published. The correct **Funding** statement reads:

The author(s) declare that financial support was received for the research and/or publication of this article. This project was supported by the Clinical Skills Enhancement Programme for Young Healthcare Professionals of the Second Hospital of Dalian Medical University (No. 2024LCJSYL20) and the In-hospital cultivation project of the Second Hospital of Dalian Medical University (No. 1).

The authors apologize for this error and state that they do not change the scientific conclusions of the article in any way. The original version of this article has been updated.

