# Supplementary material for: Global, regional, and national burden of laryngeal cancer in middle-aged and older adults from 1990 to 2021: an analysis of age and sex differences and attributable risk factors
Source: Front Public Health. 2025 May 30;13:1601029. doi: 10.3389/fpubh.2025.1601029 (PMC12162519; doi:10.3389/fpubh.2025.1601029)
Supplement: Supplementary file 1 [file Supplementary_file_1.docx]

**Supplementary figures**

- **Figure S1**. EAPC of prevalence rate of LC among MAOP for both sexes across 204 countries from 1990 to 2021. EAPC: Estimated Annual Percentage Change, MAOP: middle-aged and older populations, LC: Laryngeal cancer
- **Figure S2**. The incidence rate of LC among MAOP for both sexes across 204 countries from 1990 to 2021. MAOP: middle-aged and older populations, LC: Laryngeal cancer
- **Figure S3.** The prevalence rate of LC among MAOP for both sexes across 204 countries from 1990 to 2021. MAOP: middle-aged and older populations, LC: Laryngeal cancer
- **Figure S4.** The death rate of LC among MAOP for both sexes across 204 countries from 1990 to 2021. EAPC: Estimated Annual Percentage Change, MAOP: middle-aged and older populations, LC: Laryngeal cancer
- **Figure S5.** The DALYs rate of LC among MAOP for both sexes across 204 countries from 1990 to 2021. MAOP: middle-aged and older populations, LC: Laryngeal cancer, DALYs：Disability-adjusted life-year
- **Figure S6.** Burden of LC among MAOP by 5 SDI regions, gender, and age groups for the rate of incidence, death, and DALYs. DALYs：Disability-adjusted life-year, MAOP: middle-aged and older populations, LC: Laryngeal cancer
- **Figure S7.** The trend in prevalence rate of LC among MAOP in 21 GBD regions by SDI, 1990–2021. GBD: Global Burden of Disease，SDI: Socio-Demographic Index, MAOP: middle-aged and older populations, LC: Laryngeal cancer
- **Figure S8.** The trend in incidence rate of LC among MAOP in 21 GBD regions by SDI, 1990–2021. GBD: Global Burden of Disease, SDI: Socio-Demographic Index, MAOP: middle-aged and older populations, LC: Laryngeal cancer
- **Figure S9.** The trend in death rate of LC among MAOP in 21 GBD regions by SDI, 1990–2021. GBD: Global Burden of Disease, SDI: Socio-Demographic Index, MAOP: middle-aged and older populations, LC: Laryngeal cancer
- **Figure S10.** The trend in DALYs rate(D) of LC among MAOP in 21 GBD regions by SDI, 1990–2021. DALYs: Disability-adjusted life-year，GBD: Global Burden of Disease, SDI: Socio-Demographic Index, MAOP: middle-aged and older populations, LC: Laryngeal cancer
- **Figure S11**. Temporal trends of mortality and DALY rates attributed to alcohol use of LC among MAOP globally and in five SDI regions. Death rate attributable to alcohol use in male (A) and female (B). DALYs rate attributable to alcohol use in male (C) and female (D). MAOP: middle-aged and older populations, LC: Laryngeal cancer , DALYs：Disability-adjusted life-year
- **Figure S12.** Temporal trends of mortality and DALY rates attributed to occupational factors of LC among MAOP globally and in five SDI regions. Death rate attributable to occupational factors in male (A) and female (B). DALYs rate attributable to occupational factors in male (C) and female (D). MAOP: middle-aged and older populations, LC: Laryngeal cancer , DALYs：Disability-adjusted life-year


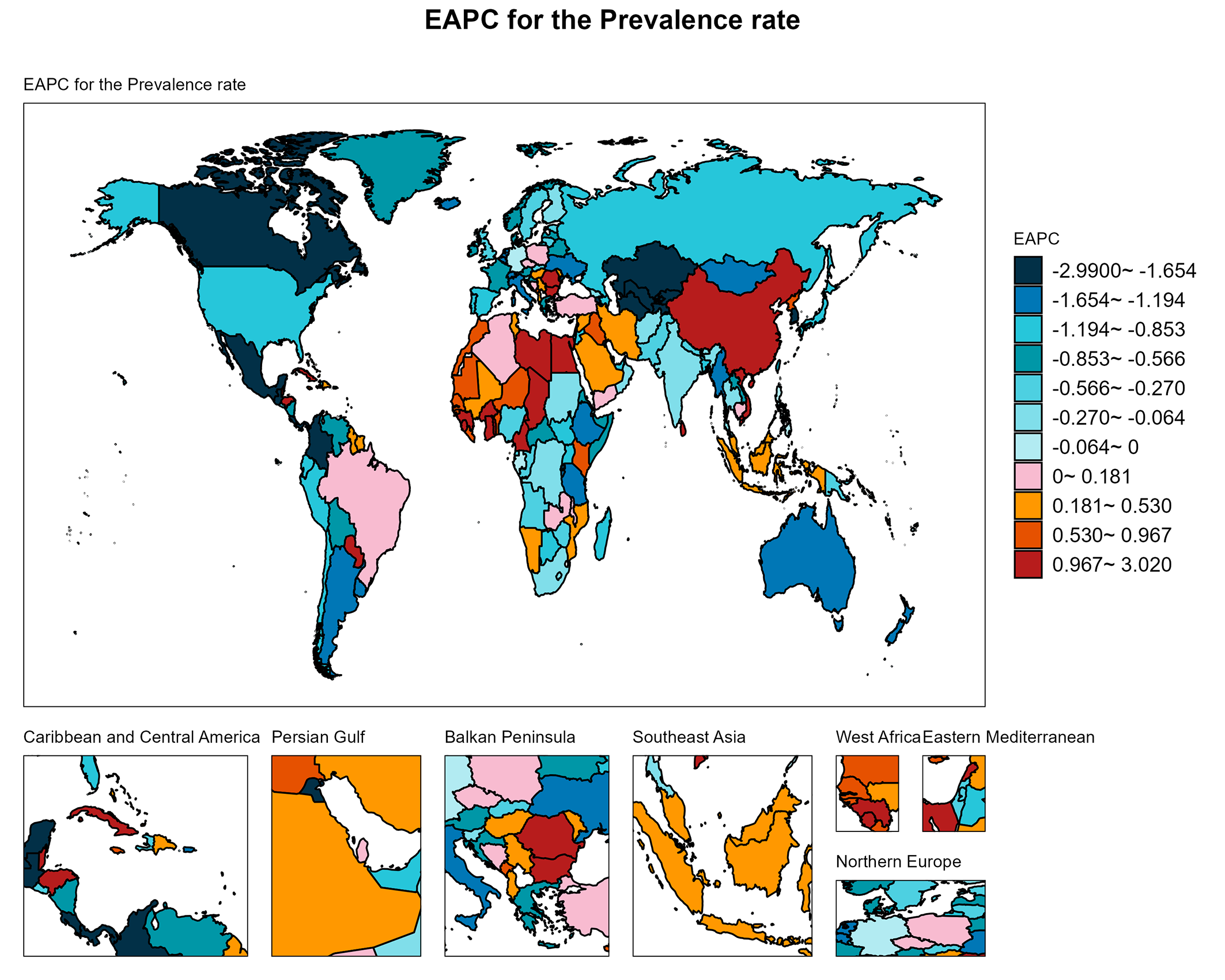


Figure S1. EAPC of prevalence rate of LC among MAOP for both sexes across 204 countries from 1990 to 2021. EAPC： Estimated Annual Percentage Change, MAOP: middle-aged and older populations, LC: Laryngeal cancer


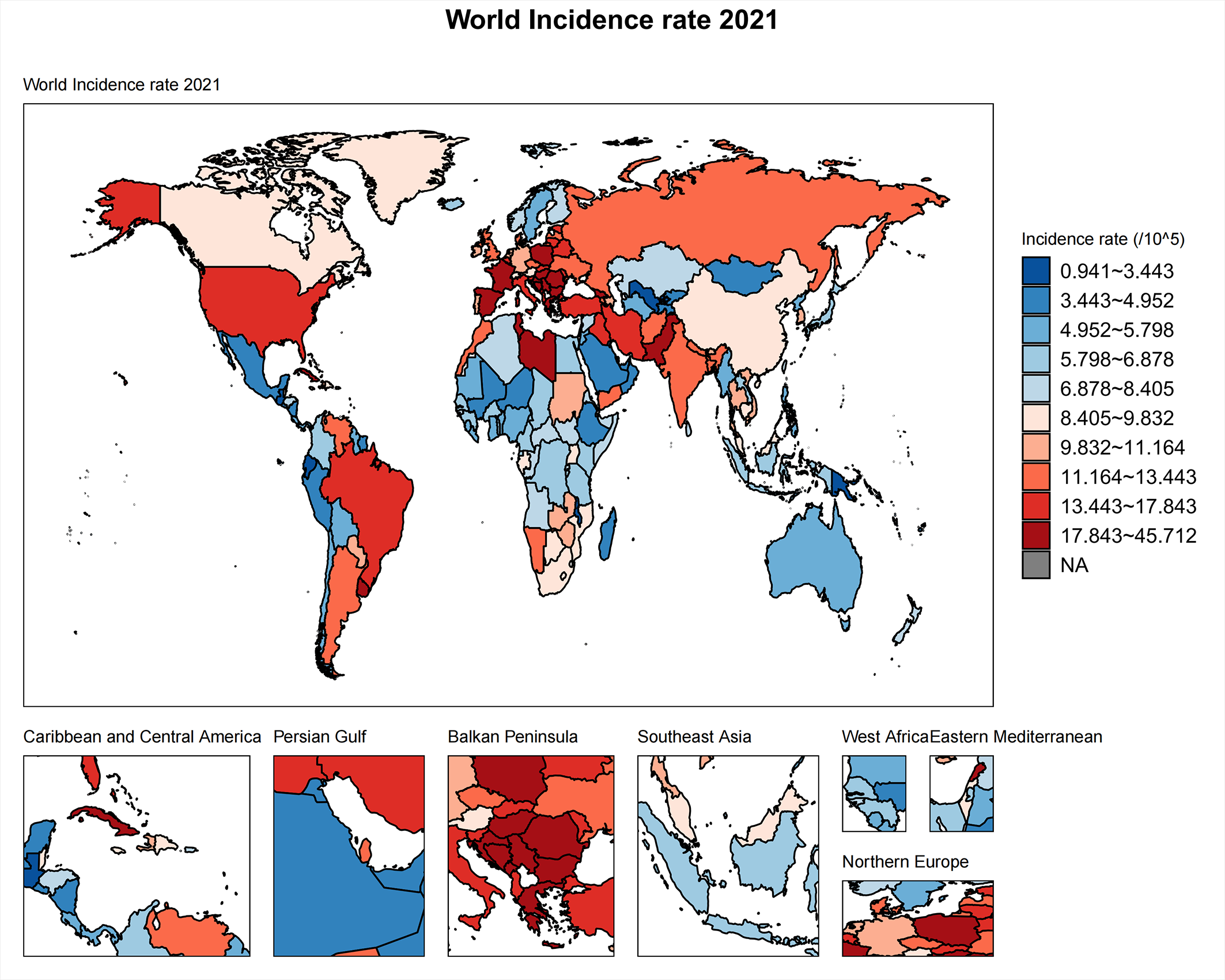


Figure S2. The incidence rate of LC among MAOP for both sexes across 204 countries from 1990 to 2021. MAOP: middle-aged and older populations, LC: Laryngeal cancer


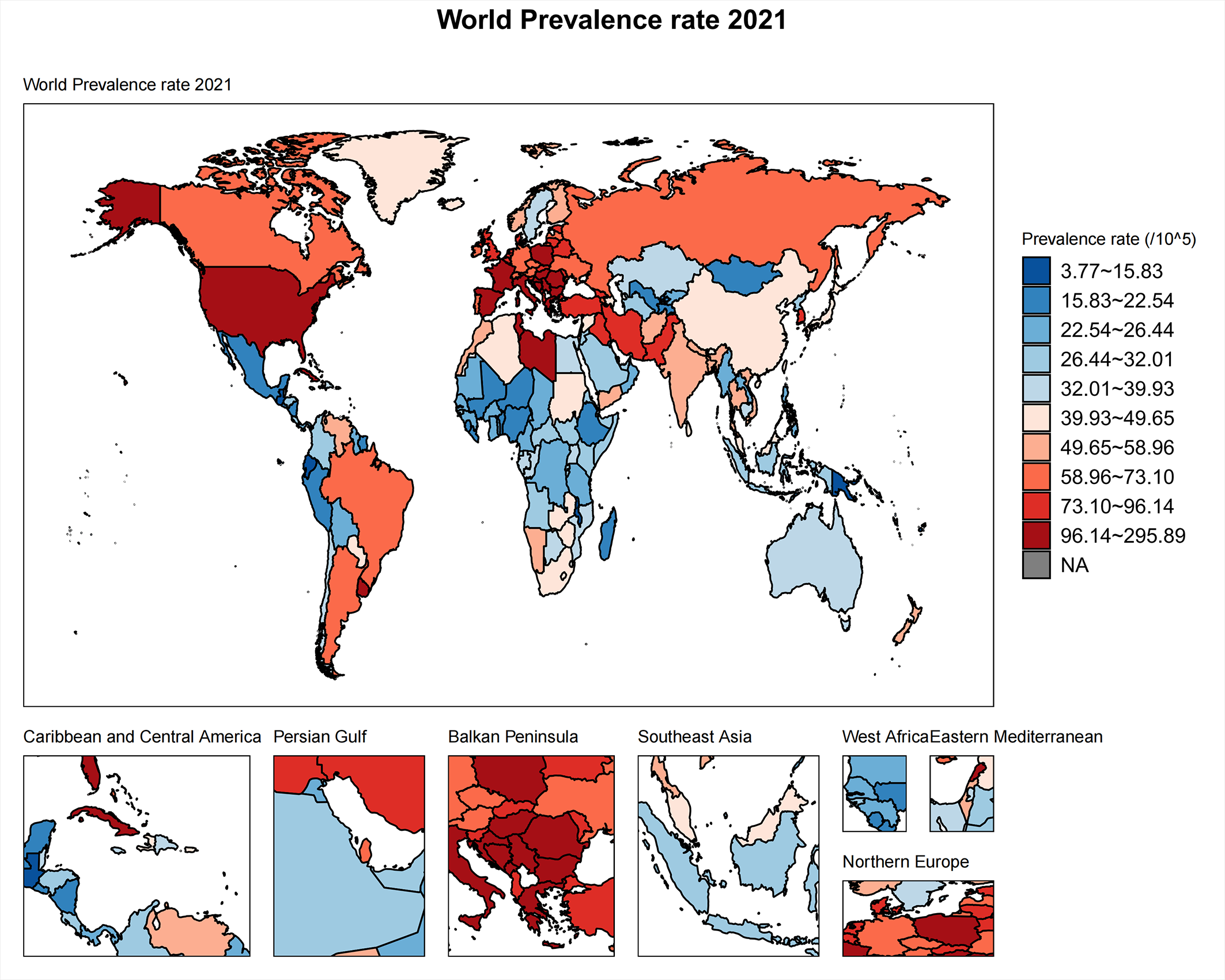


Figure S3. The prevalence rate of LC among MAOP for both sexes across 204 countries from 1990 to 2021. MAOP: middle-aged and older populations, LC: Laryngeal cancer


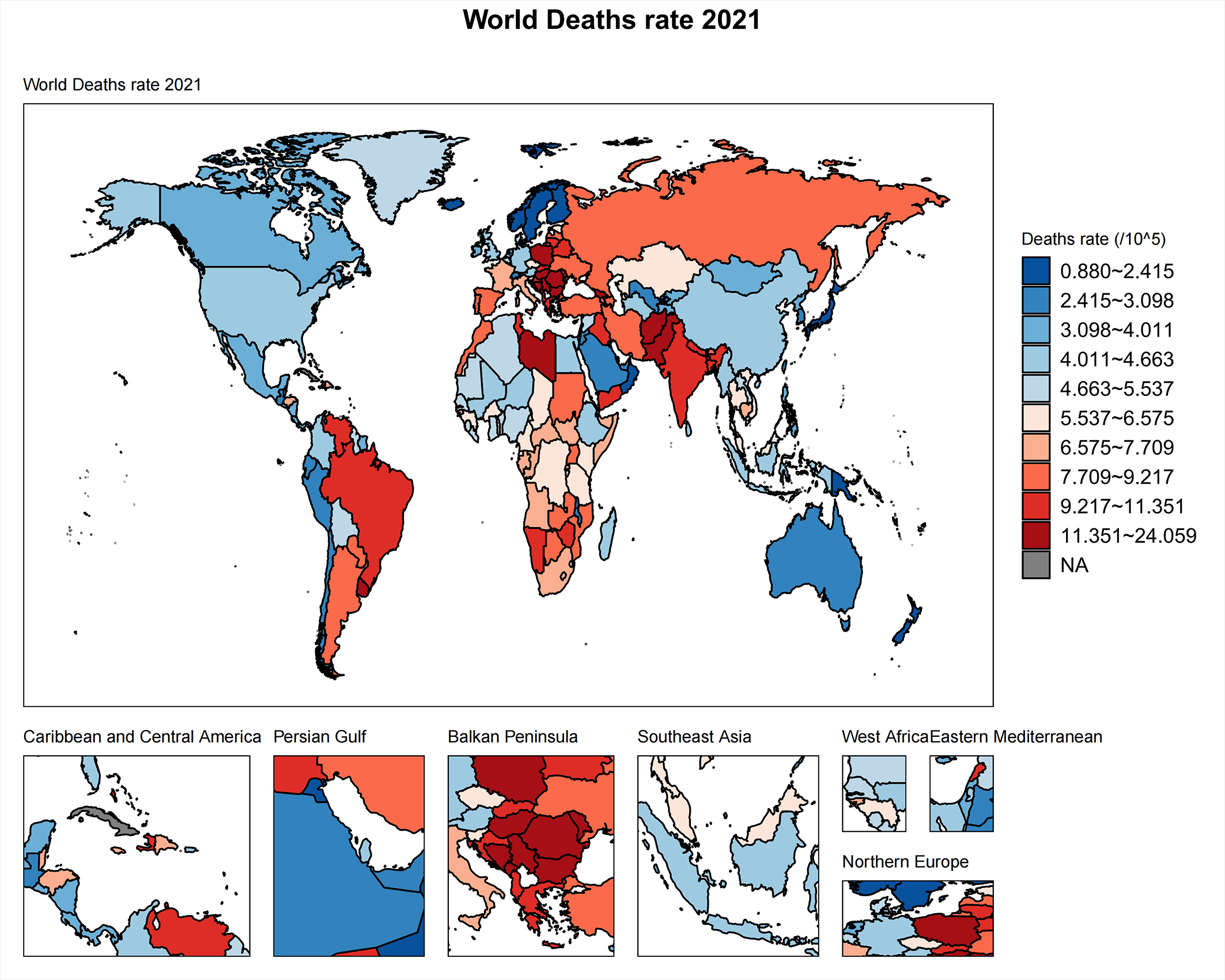


Figure S4. The death rate of LC among MAOP for both sexes across 204 countries from 1990 to 2021. MAOP: middle-aged and older populations, LC: Laryngeal cancer


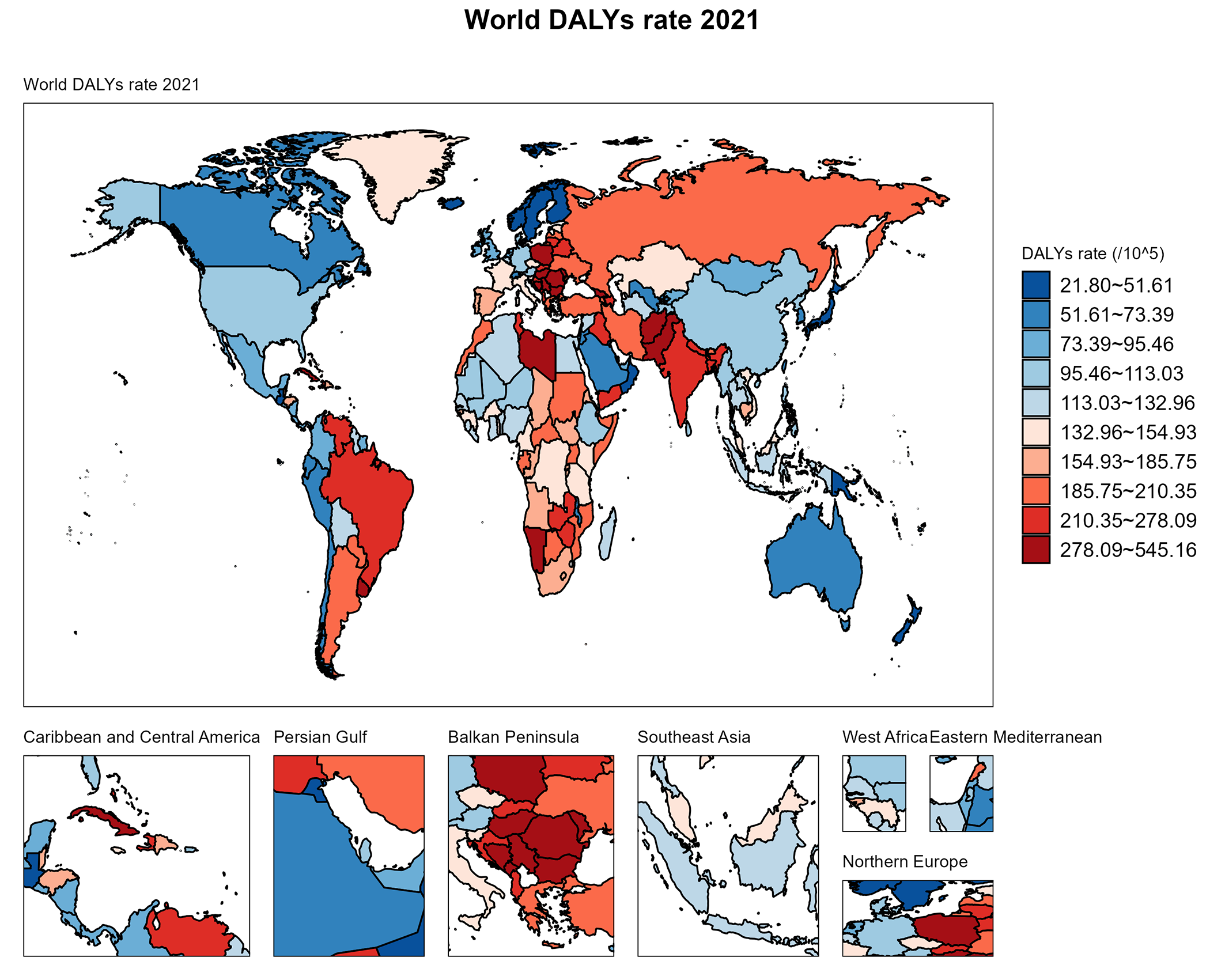


Figure S5. The DALYs rate of LC among MAOP for both sexes across 204 countries from 1990 to 2021. MAOP: middle-aged and older populations, LC: Laryngeal cancer, DALYs：Disability-adjusted life-year


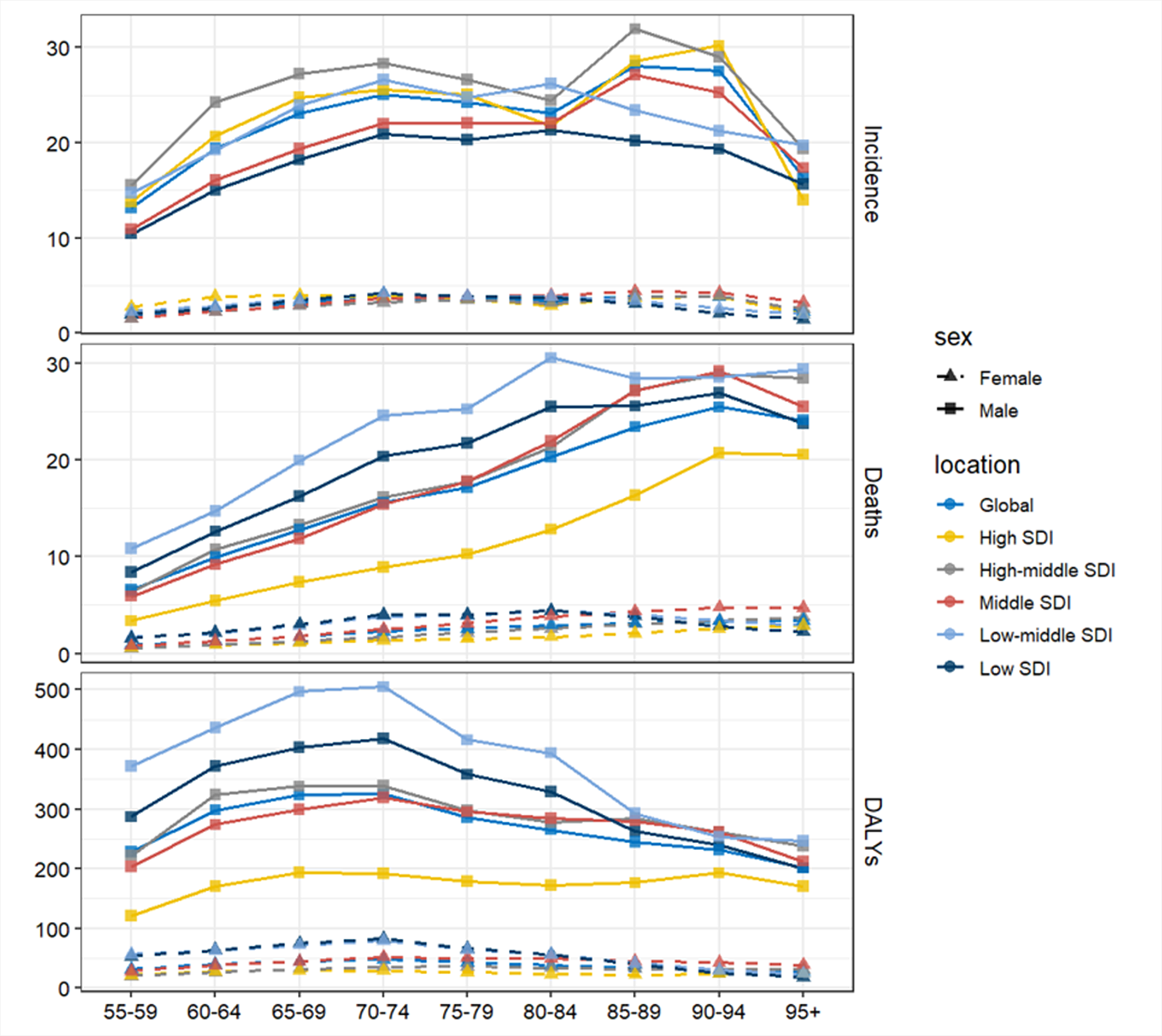


Figure S6. Burden of LC among MAOP by 5 SDI regions, gender, and age groups for the rate of incidence, death, and DALYs. DALYs：Disability-adjusted life-year, MAOP: middle-aged and older populations, LC: Laryngeal cancer





Figure S7. The trend in prevalence rate of LC among MAOP in 21 GBD regions by SDI, 1990–2021. GBD: Global Burden of Disease，SDI: Socio-Demographic Index, MAOP: middle-aged and older populations, LC: Laryngeal cancer





Figure S8. The trend in incidence rate of LC among MAOP in 21 GBD regions by SDI, 1990–2021. GBD: Global Burden of Disease, SDI: Socio-Demographic Index, MAOP: middle-aged and older populations, LC: Laryngeal cancer





Figure S9. The trend in death rate of LC among MAOP in 21 GBD regions by SDI, 1990–2021. GBD: Global Burden of Disease, SDI: Socio-Demographic Index, MAOP: middle-aged and older populations, LC: Laryngeal cancer





Figure S10. The trend in DALYs rate(D) of LC among MAOP in 21 GBD regions by SDI, 1990–2021. DALYs: Disability-adjusted life-year，GBD: Global Burden of Disease, SDI: Socio-Demographic Index, MAOP: middle-aged and older populations, LC: Laryngeal cancer


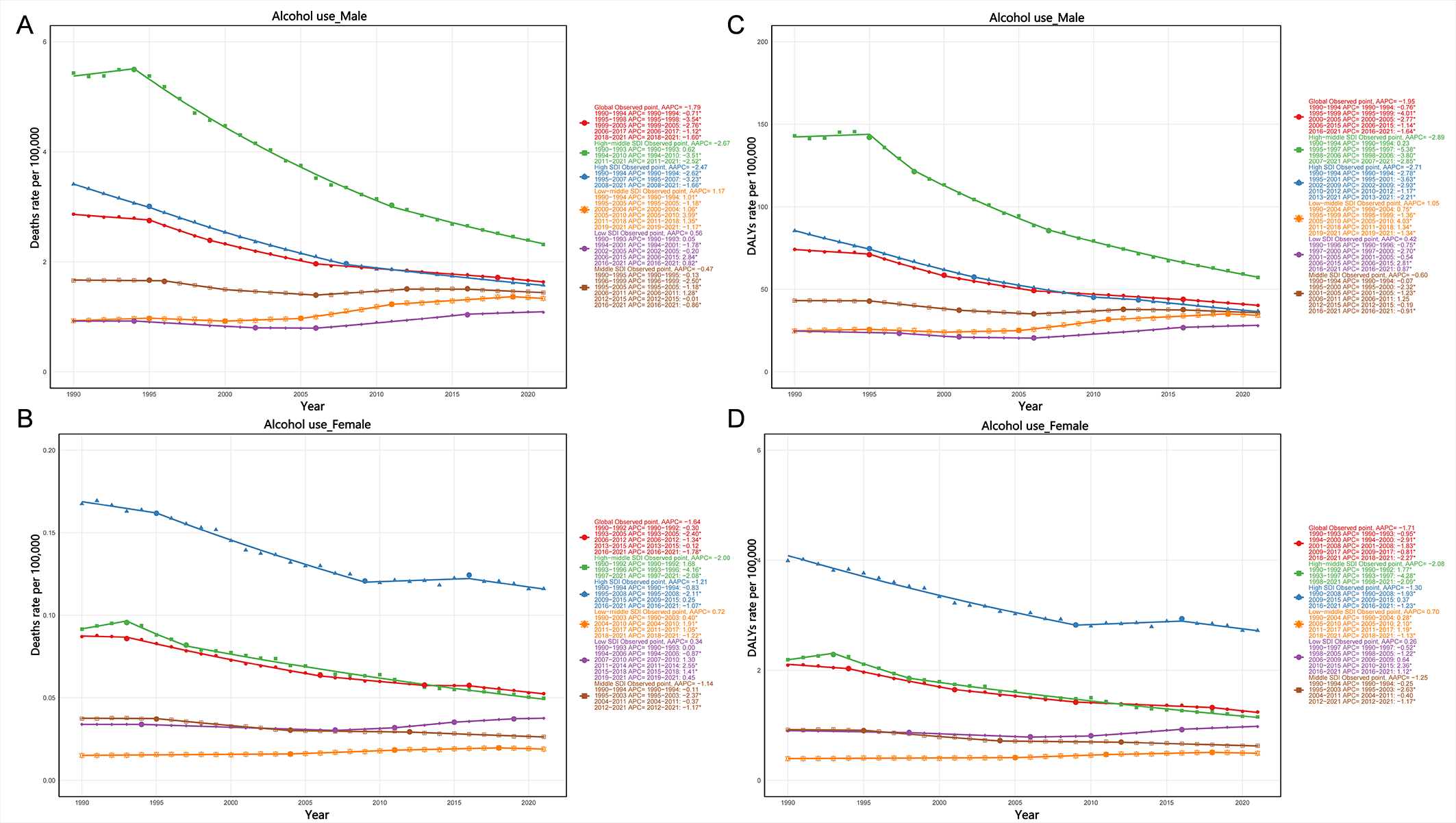


Figure S11. Temporal trends of mortality and DALY rates attributed to alcohol use of LC among MAOP globally and in five SDI regions. Death rate attributable to alcohol use in male (A) and female (B). DALYs rate attributable to alcohol use in male (C) and female (D). MAOP: middle-aged and older populations, LC: Laryngeal cancer , DALYs：Disability-adjusted life-year


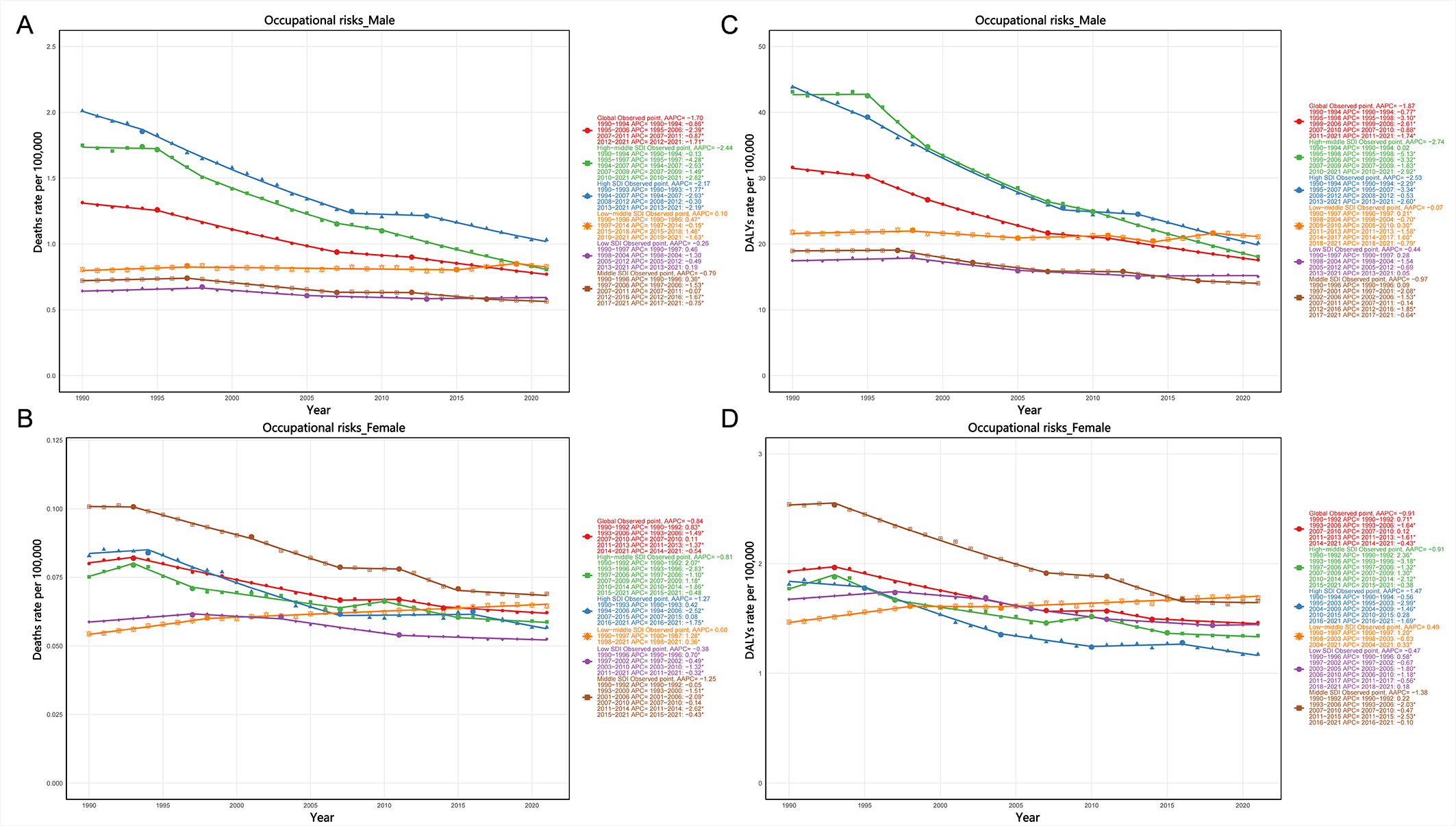


Figure S12. Temporal trends of mortality and DALY rates attributed to occupational factors of LC among MAOP globally and in five SDI regions. Death rate attributable to occupational factors in male (A) and female (B). DALYs rate attributable to occupational factors in male (C) and female (D). MAOP: middle-aged and older populations, LC: Laryngeal cancer , DALYs：Disability-adjusted life-year
